# Effects of Three-Month Administration of High-Saturated Fat Diet and High-Polyunsaturated Fat Diets with Different Linoleic Acid (LA, C18:2n–6) to α-Linolenic Acid (ALA, C18:3n–3) Ratio on the Mouse Liver Proteome

**DOI:** 10.3390/nu13051678

**Published:** 2021-05-15

**Authors:** Kamila P. Liput, Adam Lepczyński, Agata Nawrocka, Ewa Poławska, Magdalena Ogłuszka, Aneta Jończy, Weronika Grzybek, Michał Liput, Agnieszka Szostak, Paweł Urbański, Agnieszka Roszczyk, Chandra S. Pareek, Mariusz Pierzchała

**Affiliations:** 1 Department of Genomics and Biodiversity, Institute of Genetics and Animal Biotechnology of the Polish Academy of Sciences, ul. Postepu 36A, Jastrzebiec, 05-552 Magdalenka, Poland; k.liput@igbzpan.pl (K.P.L.); a.nawrocka@igbzpan.pl (A.N.); e.polawska@igbzpan.pl (E.P.); m.ogluszka@igbzpan.pl (M.O.); a.szostak@igbzpan.pl (A.S.); p.urbanski@igbzpan.pl (P.U.); a.roszczyk@igbzpan.pl (A.R.); 2 Department of Molecular Biology, Institute of Genetics and Animal Biotechnology of the Polish Academy of Sciences, ul. Postepu 36A, Jastrzebiec, 05-552 Magdalenka, Poland; a.jonczy@igbzpan.pl; 3 Department of Physiology, Cytobiology and Proteomics, West Pomeranian University of Technology, K. Janickiego 32 Str., 71-270 Szczecin, Poland; Adam.Lepczynski@zut.edu.pl; 4 Department of Experimental Genomics, Institute of Genetics and Animal Biotechnology of the Polish Academy of Sciences, ul. Postepu 36A, Jastrzebiec, 05-552 Magdalenka, Poland; 5 Department of Biotechnology and Nutrigenomics, Institute of Genetics and Animal Biotechnology of the Polish Academy of Sciences, ul. Postepu 36A, Jastrzebiec, 05-552 Magdalenka, Poland; w.grzybek@igbzpan.pl; 6 Department of Stem Cell Bioengineering, Mossakowski Medical Research Institute of the Polish Academy of Sciences, 02-106 Warsaw, Poland; mliput@imdik.pan.pl; 7 Institute of Veterinary Medicine, Faculty of Biological and Veterinary Sciences, Nicolaus Copernicus University, 87-100 Toruń, Poland; pareekcs@umk.pl; 8 Division of Functional Genomics in Biological and Biomedical Research, Centre for Modern Interdisciplinary Technologies, Nicolaus Copernicus University, 87-100 Torun, Poland

**Keywords:** HFD, SFA, omega-3 PUFA, omega-6 PUFA, nutriproteomics, 2DE, MALDI TOF/TOF

## Abstract

The aim of the study was to evaluate the effect of different types of high-fat diets (HFDs) on the proteomic profile of mouse liver. The analysis included four dietary groups of mice fed a standard diet (STD group), a high-fat diet rich in SFAs (SFA group), and high-fat diets dominated by PUFAs with linoleic acid (LA, C18:2n–6) to α-linolenic acid (ALA, C18:3n–3) ratios of 14:1 (14:1 group) and 5:1 (5:1 group). After three months of diets, liver proteins were resolved by two-dimensional gel electrophoresis (2DE) using 17 cm non-linear 3–10 pH gradient strips. Protein spots with different expression were identified by MALDI-TOF/TOF. The expression of 13 liver proteins was changed in the SFA group compared to the STD group (↓: ALB, APOA1, IVD, MAT1A, OAT and PHB; ↑: ALDH1L1, UniProtKB—Q91V76, GALK1, GPD1, HMGCS2, KHK and TKFC). Eleven proteins with altered expression were recorded in the 14:1 group compared to the SFA group (↓: ARG1, FTL1, GPD1, HGD, HMGCS2 and MAT1A; ↑: APOA1, CA3, GLO1, HDHD3 and IVD). The expression of 11 proteins was altered in the 5:1 group compared to the SFA group (↓: ATP5F1B, FTL1, GALK1, HGD, HSPA9, HSPD1, PC and TKFC; ↑: ACAT2, CA3 and GSTP1). High-PUFA diets significantly affected the expression of proteins involved in, e.g., carbohydrate metabolism, and had varying effects on plasma total cholesterol and glucose levels. The outcomes of this study revealed crucial liver proteins affected by different high-fat diets.

## 1. Introduction

Saturated fatty acids (SFAs) are mainly found in dairy foods, lards, processed meats—e.g., salami, sausage and bacon—manufactured foods—including snacks, cakes and biscuits—and deep-fried foods. Plant-derived oils like palm and coconut oils are also sources of SFAs [1]. In recent years, interest in coconut oil consumption has increased. For instance, this oil consumption in the USA increased by 34% in 2014 compared to 2004. The reason for such a popularity of coconut oil, particularly cold-pressed, was mostly due to information about its health-promoting properties, such as anti-proliferative and pro-apoptotic effects on breast cancer cells of the dominant SFA in coconut oil—lauric acid (12:0) [2]. Additionally, the high content of medium-chain triacylglycerols (MCTs) was mentioned as one of the arguments for coconut oil consumption [3]. Scientific reports have indicated that MCTs induced thermogenesis and reduced food consumption [4,5]. However, this evidence was insufficient to attribute MCT properties to coconut oil in weight loss support.

The highly publicized issue of coconut oil properties, which assist in reducing body weight, was not supported by scientific evidence [6]. Moreover, dietary recommendations encourage limiting SFA intake and replacing them with polyunsaturated fatty acids (PUFAs) to decrease the risk of metabolic disorders, for example, the World Health Organization and American Dietary Guidelines 2015–2020 recommend reducing SFA intake to less than 10 percent of calories per day [7]. The American Heart Association stated that reducing SFAs intake to 5–6% of daily calories had a beneficial effect on adults by lowering LDL-C [8]. An epidemiological study by Kromhout et al. found a positive correlation between SFA consumption and coronary heart disease incidence (CHD) [9]. In contrast, a diet rich in high-unsaturated fatty acids may decrease risk factors, morbidity, and mortality related to coronary vascular disease (CVD) [10]. The recommendations for replacing saturated and trans fats with unsaturated fats were also supported by the results describing a reduction in total mortality associated with replacing 5% of energy from saturated fats with equivalent energy derived from polyunsaturated and monounsaturated fats [11]. According to Gouaref et al., increased pro-inflammatory cytokine concentrations (TNFα, IL-6, IL1β) were associated with elevated levels of saturated non-esterified fatty acids (NEFAs) and decreased plasma PUFAs. A positive correlation was also found between the plasma PUFA/SFA and HDL-c/LDL-c ratios (r = +66, *p* < 0.01) in the group of patients with type 2 diabetes mellitus (T2DM) and hypertension [12]. Additionally, female mortality caused by breast cancer was the highest in the group characterized by high saturated fat intake [1,13].

The Mediterranean dietary pattern (MedDiet) and Dietary Approaches to Stop Hypertension (DASH) dietary pattern are the most frequently studied dietary recommendations for preventing and treating heart failure. Both share the common characteristic of restricting saturated fatty acids than the Westernized dietary pattern, specifically to a diet with high saturated fatty acids. Additionally, the MedDiet emphasized increased consumption of unsaturated fatty acids (UFA), which are composed of both monounsaturated (MUFA) and polyunsaturated fatty acids (PUFA) found in fatty fish and plant oils [14].

However, recommendations to reduce saturated fatty acid consumption are currently questioned because of inconclusive evidence that dietary saturated fats are associated with an increased risk of CHD or CVD [15]. Despite many studies, the role of dietary SFAs and PUFAs in metabolic disturbances remains unclear. According to Uger at al. consolidation all dietary SFAs into a single group was an oversimplification and the SFAs effects on health should be considered regarding the chemical structure and dietary source [16].

Thus, the present work aimed to identify key hepatic protein mediators involved in biological mechanisms of action of different high-fat diets based on plant-derived oils with varying SFA and PUFA contents.

## 2. Materials and Methods

### 2.1. Animals, Diets and Sampling

The nutritional experiment was carried out in the animal house of the Institute of Genetics and Animal Biotechnology of the Polish Academy of Sciences in Jastrzębiec. Experimental procedures were approved by the Second Warsaw Local Ethics Committee for Animal Experimentation (WAW2_22/2016). Animals were kept in standard cages under temperature- and humidity-controlled conditions with a 12-h light/dark cycle.

Male Swiss-Webster mice (*n* = 32) were fed standard growth diets for eight weeks after weaning. Next, animals were separated into four groups (8 mice per group). Mice were fed one of the four types of feed. The first group (STD group) were mice fed complete chow for laboratory animals containing 22% crude protein, 4.2% crude fat, 3.5% crude fiber, 5.7% crude ash (Labofeed H, “Morawski” Feed Factory, Kcynia, Poland). The second group (SFA group) were mice fed Labofeed H with coconut virgin oil. The third and the fourth groups were mixtures of Labofeed H feed with the addition of vegetable oils with high PUFAs content, varying in the ratio of linoleic acid (18:2n–6, LA) to α-linolenic acid (18:3n–3, ALA), in details, the first group ratio omega-6/3 was 13.76:1 (14:1 group) and in the second was 5:1 (5:1 group). The composition and the fatty acid contents in each diet were varied using different oils addition according to Table 1.

The prepared diets were vacuum packed, stored in the dark and given to the animals two times per day to avoid fatty acid oxidation. The mice had ad libitum access to water and food and were weighed every two weeks. After three months of feeding with specific diets, animals were sacrificed, livers were dissected out, and median lobes were frozen in liquid nitrogen and then stored at −70 °C for further analysis.

The amounts of added vegetable oils were determined based on the analysis of the fatty acid content of individual oils using a GC-7890 gas chromatograph (Agilent Technologies, Inc., Santa Clara, CA, USA) with a flame ionization detector (FID) and a 60 m capillary column, 0.25 mm internal diameter and 0.20 μm stationary layer thickness (Hewlett-Packard-88, Agilent J&W GC Columns, Santa Clara, CA, USA). The carrier gas was helium with a flow rate of 50 mL/min. The dispenser and detector temperatures were 260 °C. Temperature program: (1) from 140 °C to 190 °C (4 °C/min), (2) from 190 °C to 215 °C (0.8 °C/min). The results were compared to the external standard Supelco 37 Component FAME Mix, 47885-U, (Sigma-Aldrich Sp. z o.o., Poznan, Polandrimental diets according to [17].

### 2.2. Plasma Biochemical Parameters

Blood was collected after death by cardiac puncture and mixed with 10 μL 0.5M EDTA and centrifuged at 3000× *g* for 10 min at 4 °C. Next, plasma was collected and frozen at −70 °C. Biochemical analyses were performed using COBAS INTEGRA^®^ 400 plus system (Roche Diagnostics Ltd., Rotkreuz, Switzerland). Following biochemical tests were used: ALB2 (albumin); ALTL (alanine aminotransferase), ASTL (aspartate aminotransferase), BILT3 (total bilirubin), CHE2 (cholinesterase), CHOL2 (total cholesterol), GLUC3 (glucose), HDLC3 (high-density lipoprotein-cholesterol), IRON2 (iron), LDL_C (low-density lipoprotein-cholesterol), LIPC (lipase), TP2 (total protein), and TRIGL (triacylglycerols).

### 2.3. Two-Dimensional Electrophoresis

#### 2.3.1. Homogenization

Livers were dissected out and perfused by ice-cold PBS and stored at −70 °C. About 100 mg of the medial lobe of mouse liver was added to 2 mL vials containing ceramic beads (1.4 mm of diameter) and lysis buffer: 7 M urea, 2 M thiourea, 4% *w*/*v* CHAPS and protease inhibitors (cOmplete™, Mini Protease Inhibitor Cocktail, Roche Diagnostics GmbH, Mannheim, Germany) and then homogenized using MagNA Lyser Instrument (Roche Diagnostics GmbH, Mannheim, Germany). The homogenization conditions were as follows: two runs of 20 s at 4000 rpm and one run of 20 s at 5000 rpm (between each run samples were put in the cooling block for 3 min). Subsequently, the homogenates were centrifuged at 12,000× *g* for 25 min at 4 °C. Supernatants were collected and stored at −70 °C.

#### 2.3.2. First Dimension—Isoelectrofocusing (IEF)

A modified Bradford assay was used to determine the protein extract’s concentration according to the manufacturer’s instructions (Protein Assay Dye Reagent Concentrate; Bio-Rad Laboratories, Inc., Hercules, CA, USA). The standard curve was determined from the series standard dilution of bovine serum albumin (BSA). Samples containing 660 μg of total protein were mixed with lysis buffer (7 M urea, 2 M thiourea, 4% CHAPS, 1% (*w*/*v*) dithiothreitol (DTT) and 0.5% (*v*/*v*) ampholytes (40% BioLyte^®^ 3/10 Ampholyte, Bio-Rad Laboratories, Inc., Hercules, CA, USA) to adjust to a final volume of 330 μL per sample. Each liver sample was analyzed in two technical replicates. The precast immobilized pH gradient strips (non-linear pH 3–10, 17-cm length) were loaded with 600 μg of proteins by in-gel rehydration (passive—5 h, 0 V and active—12 h, 50 V). After rehydration, one electrode wick with 20 μL 0.01% DTT was placed in the cathode, and the other electrode wick with water was placed in the anode. Focusing began at 250 V for 125 Vh, 500 V for 250 Vh, 1000 V for 500 Vh in rapid mode, 5000 V for 1.5 h in linear mode and 5000 V for 90,000 Vh in rapid mode. During rehydration and IEF IPG strips were overlaid with mineral oil. Rehydration and isoelectrofocusing were carried in Protean i12 IEF Cell (Bio-Rad Laboratories, Inc., Hercules, CA, USA) at 20 °C. The separation of proteins in the non-linear range of pH 3–10 was assessed using the two-dimensional standard (2-D SDS-PAGE Standards, Bio-Rad Laboratories, Inc., Hercules, CA, USA). The mixture contained the following proteins with different molecular masses and isoelectric points conalbumin (76 kDa, pI 6.0, 6.3, 6.6), bovine serum albumin (66.2 kDa, pI 5.4, 5.6), actin (43 kDa, pI 5.0, 5.1), GAPDH (36 kDa, pI 8.3, 8.5), carbonic anhydrase (31 kDa, pI 5.9, 6.0), trypsin inhibitor (21.5 kDa, pI 4.5) and myoglobin (17.5 kDa, pI 7.0).

#### 2.3.3. Second Dimension—SDS-PAGE

After IEF, the IPG strips were stored at −70 °C and prior to separation on the second dimension were transferred into equilibration buffer (6 M urea, 2% *w*/*v* SDS, 30% *v*/*v* glycerol, 17 mM Tris-HCl pH 6.8) for 15 min with additional 1% (*w*/*v*) DTT. For the next 20 min, IPG strips were incubated with equilibration buffer with 2.5% (*w*/*v*) iodoacetamide at room temperature. Finally, the strip was placed in 12% SDS-PAGE gel (aqueous acrylamide and bisacrylamide solution at a ratio of 29:1, 18 cm × 20 cm × 1.00 mm) and was fixed in place with a 0.5% (*w*/*v*) low-melting agarose overlay containing a trace amount of bromophenol blue to track electrophoresis. Gels were run in PROTEAN xi cell (Bio-Rad Laboratories, Inc., Hercules, CA, USA) 40 V for 2.5 h and 100 V for 18 h. The running buffer (25 mM Tris, 192 mM glycine, 0.1% SDS) was cooled externally to 10 °C. The size standard used to determine the molecular weight on 2DE gels contained the following masses of proteins: 250 kDa, 150 kDa, 100 kDa, 75 kDa, 50 kDa, 37 kDa, 25 kDa, 20 kDa, 15 kDa and 10 kDa (Precision Plus Protein^TM^ Standard Plugs, Unstained, Bio-Rad Laboratories, Inc., Hercules, CA, USA).

#### 2.3.4. Image Staining and Analysis

After SDS-PAGE, the gels were washed in 500 mL distilled water three times. The gels were stained according to protocols described by [18,19]. Gels were fixed three hours in 750 mL buffer containing: 28.8% ethanol, 2.6% phosphoric acid and distilled water. Next, gels were incubated in staining buffer composed of 5% ammonium sulfate-18-hydrate, 10% ethanol, 0.02% Coomassie G-250 and 8.5% phosphoric acid for three hours. Gels were washed in 200 mL distilled water and destained using a solution of 10% ethanol and 2% phosphoric acid.

After staining, gels were scanned on a GelDoc XR+ (Bio-Rad Laboratories, Inc., Hercules, CA, USA). Protein spots were assigned automatically and manually verified. Protein spots densitometric analyses were performed by the PDQuest™ Advanced 8.0.1 software (Bio-Rad Laboratories, Inc., Hercules, CA, USA), applying the local regression model (LOESS) to normalize spot intensity.

The average value of the protein spot’s densitometric intensity was applied to estimate changes between the groups. The fold of change below 1.00 means a decrease, whereas above 1.00 means the protein level increase. Based on the fold change value, a heat map was prepared in GraphPad Prism 7.04 (GraphPad Software, San Diego, CA, USA). Assignments of differentially expressed proteins according to their participation in metabolic pathways were performed using STRING 11.0 and the following databases: KEGG, REACTOME and Gene Ontology [20].

### 2.4. Matrix-Assisted Laser Desorption Ionization–Time of Flight Mass Spectrometry (MALDI-TOF MS)

The significantly differentiated protein spots have been analyzed using matrix-assisted laser desorption/ionization time-of-flight mass spectrometry (MALDI-TOF MS) to determine protein mass fingerprint and identify the specific protein. Biological replicates of each protein spots were manually excised from the average three polyacrylamide gels. Then, excised spots were destained with a solution of 200 mM NH_4_HCO_3_ in 40% acetonitrile and incubated at 37 °C for 30 min. Next, spots were vacuum-dried for 15–25 min. The dry gel piece was incubated for 16 h in 20 μL 20 μg/mL trypsin (0.4 μg trypsin in 40 mM NH_4_HCO_3_, 9% acetonitrile, 1 mM HCl per one spot) at 37 °C. Digestion was terminated by the addition of 2 µL of 5% trifluoroacetic acid (TFA). Peptide-matrix crystal layer was obtained on 800 µm AnchorChip 384 BC target plate (Bruker Daltonik GmbH, Bremen, Germany). Briefly, 1 µL of peptides from the in-gel digestions supernatant was loaded on the target plate and left to dry completely. Then, 1 µL of 5 μg/μL CHCA matrix solution (α-cyano-4-hydroxycinnamic acid in 70% acetonitrile and 0.1% TFA) was applied to the same spot and left to dry completely. Mass spectra were acquired in positive-ion reflector mode using the ultrafleXtreme^TM^ spectrometer (Bruker Daltonik GmbH, Bremen, Germany). Peptide Calibration Standards Mix II (Bruker Daltonik GmbH, Bremen, Germany) was used for external calibration of the mass scale. Typically, 2000 shots were accumulated for mass spectrum generation in MS mode. The peak lists were cleared from all background peaks (such as matrix peaks—generated from CHCA crystallines, keratin contamination or polyacrylamide—from gel piece digestion without visible spot). The spectra were processed using the FlexAnalysis 3.4 and BioTools 3.2. Peptide alignment and protein identification was performed using the Peptide Mass Fingerprinting (PMF) technique. Data were compared to the mammalian SwissProt database using MASCOT software (http://www.matrixscience.com/ (accessed on 13 May 2021)). The following parameters were used for database searches: (1) trypsin digestion; (2) cysteine carbamidomethylation (Carbamidomethyl (C)) as a fixed modification; (3) acetylation (Acetyl (Protein N-term) and methionine oxidation (Oxidation (M) as variable modifications; (4) mass tolerance to 150 ppm; and (5) maximum one missed cleavage site. The theoretical molecular mass and theoretical isoelectric point of statistically significant hits were compared with the localization of their protein spots in 2DE gel following two protein standards: one-dimensional Precision Plus Protein^TM^ Standard Plug (Bio-Rad Laboratories, Inc., Hercules, CA, USA) and two-dimensional 2-D SDS-PAGE Standards (Bio-Rad Laboratories, Inc., Hercules, CA, USA). UniProt [21], Gene Ontology [22,23] and pLoc-mEuk [24] were used to determine the cellular location of identified proteins.

### 2.5. Protein Expression Analysis by Western Blotting

Total protein was extracted from approximately 30 mg of liver samples using AllPrep RNA/DNA/PROTEIN (QIAGEN) according to the manufacturer’s instructions. The protein pellet was dissolved in lysis buffer containing 7 M urea, 2 M thiourea and 4% CHAPS. Samples were boiled in Laemmli reducing sample buffer (62.5 mM Tris-HCl pH 6.8, 20% glycerol, 2% SDS, 5% β-mercaptoethanol, bromophenol blue), separated by 12% SDS-PAGE and transferred to a PVDF membrane. Specific antibodies are listed in Table S1 in the Supplementary Materials Chemiluminescence signals were detected using Clarity Western ECL Substrate (Bio-Rad Laboratories, Inc.) and ChemiDoc XRS+ Gel Imaging System (Bio-Rad Laboratories, Inc., Hercules, CA, USA).

The band lane’s intensity value was quantified using Quantity One 1-D Analysis Software in Optical Density units (Bio-Rad Laboratories, Inc., Hercules, CA, USA). The experimental intensity values of target proteins (OAT and PRDX6) were normalized using the lane normalization factor, the value of the observed signal for GAPDH in each lane divided by the highest observed GAPDH signal on the blot. The normalized signal of each experimental target band was calculated by dividing each experimental target band’s observed signal intensities by the determined lane normalization factor.

### 2.6. RNA Extraction and Quantitative RT-PCR

#### 2.6.1. RNA Isolation

Total RNA was extracted from approximately 30 mg of liver samples from 6 samples per group using AllPrep RNA/DNA/PROTEIN (QIAGEN) according to manufacturer’s instructions with the following modifications: (1) flow-through with 96% ethanol was added to RNeasy spin column into two parts (each time 430 μL) and (2) only 680 μL RW1 was used. The yield and purity of extracted total RNA were determined by NanoDrop ND-1000 spectrophotometer (Thermo Fisher Scientific Inc., Waltham, MA, USA) RNA samples were diluted to a concentration of approx. 640.28 ng/μL. RNA samples with an absorbance ratio OD 260/280 between 2.01–2.11 (average 2.05) and OD 260/230 between 2.01–2.31 (average 2.13) were used for RT reaction. RNA integrity was confirmed using 1% agarose gel electrophoresis with 0.002% ethidium bromide in TBE buffer (0.089 M Tris base, 0.089 M boric acid, 0.0025 M EDTA). Extracted RNA mixed with gel loading solution contained 60 mM Tris-HCl (pH 7.6), 60 mM EDTA, 0.003% bromophenol blue, 0.03% xylene cyanol FF, 60% glycerol (BLIRT S.A., Gdansk, Poland) and heated for 10 min in 70 °C. The gels were run for 60 min with constant voltage (90 V) before imaging under UV transillumination using UV Gel Doc XR+ (Bio-Rad Laboratories, Inc., Hercules, CA, USA).

#### 2.6.2. Reverse Transcription (RT)

The first-strand cDNA synthesis was done using the Transcriptor First Strand cDNA Synthesis Kit (Roche Diagnostics GmbH, Mannheim, Germany). Reaction mix preparation: 1.5 μg of total RNA was reverse-transcribed using 2.5 μM anchored-oligo d(T)_18_ primers and 60 µmol random hexamers and 10 U transcriptor reverse transcriptase in a final volume 20 μL. Stages of reverse transcription: denaturation of the template-primer mix at 65 °C for 10 min, reverse transcription at 25 °C for 10 min and 50 °C for 60 min, inactivation of transcriptor reverse transcriptase at 85 °C for 5 min. In each qPCR run, non-reverse transcriptase controls (NRT) were included.

#### 2.6.3. Primers Design

Sequences of primers for reference and target genes were designed using Primer-BLAST (https://www.ncbi.nlm.nih.gov/tools/primer-blast/ (accessed on 13 May 2021)) on the genes mRNA nucleotide sequences from Gene database (https://www.ncbi.nlm.nih.gov/gene (accessed on 13 May 2021)), based on the following parameters: (1) primer length: 19–23 bp, (2) GC content—50–60%, (3) Tm—50–65 °C, (4) Max Tm difference = 3 °C, (5) Primer must span an exon-exon junction and (6) amplicon length 50–210 bp. All primers used in this study were synthesized in the Genomed S. A. The reference gene candidates were selected from the literature [25,26]. NormFinder was used to determine expression stability of selected candidate reference genes—beta-2 microglobulin (*B2m*), peptidylprolyl isomerase (*Ppia*) and cyclin G associated kinase (*Gak*) [27]. NormFinder identified *Gak* as the optimal reference gene, with the stability value equals 0.106. Sequences of all primers are provided in Supplementary Materials in Table S2.

#### 2.6.4. Amplified Products Verification and Real-Time PCR Analysis

Six biological replicates for each group and three technical replicates for *Prdx6* and *Oat* of each biological replicate were analyzed for qPCR analysis. No-template controls (with water) were run for every reaction. Real-time PCRs were conducted in the 20 μL mixture contained 5 μL of diluted cDNA (dilution factor 25), 200 nM each of the forward and reverse primers and 10 μL of LightCycler^®^ 480 SYBR Green I Mater (Roche Diagnostics GmbH, Germany). The qPCR was performed in LightCycler 480 Thermocycler (Roche Diagnostics GmbH, Germany) using the following cycle parameters: initial denaturation 95 °C, 5 min, amplification in 45 cycles of 95 °C for 10 s, 59 °C for 10 s and 72 °C for 20 s. Post-amplification melting-curve analysis was performed under conditions: 95 °C for 5 s, 65 °C for 1 min, and the temperature was increased to 97 °C.

All primer sets were tested for specificity in 2.5% agarose gel electrophoresis stained with SYBR^TM^ Safe DNA Gel Stain (Thermo Fisher Scientific Inc., Waltham, MA, USA). Amplification specificity of each reaction was done by melting curve analysis.

The quantification of gene expression by cycle (Cq) values of every sample were done by LightCycler 480 Software ver. 1.5.1.62 qPCR efficiencies in the exponential phase were calculated for each primer pair for each amplification plate using LinRegPCR version 2018.0 [28]. The relative expression levels for each mRNA transcript were based on the method described by Pfaffl M.W. (2001) [29,30]. Calibrator was the average value of the SFA group. All target genes were normalized to a reference gene (*Gak*).

### 2.7. Statistical Analysis

Alterations in mass body weights during the experiment were analyzed by Two-Way ANOVA and Tukey’s multiple comparisons test using GraphPad Prism 7.04 (GraphPad Software, San Diego, CA, USA).

The comparative densitometric analysis of protein spots included liver samples isolated from 32 mice fed a 3-month diet consisted of a set of 510 spots that occurred on each of 2DE gels. The values of each spot’s intensity in the four groups in two technical replications for individuals were used to analyze the multiple regression function recorded in the form of a model (1). The same model (1) was used to analyze biochemical and relative expression levels of selected target genes. The analysis was performed in the SAS 9.4 system. (SAS Institute Inc., Cary, NC, USA). The differences between diet groups were estimated using Tukey’s test. For the observations that presented the data distribution were non-normal, the Wilcoxon Mann–Whitney test was used to compare the differences between diet groups. The levels of significance in this model were marked as follows: *—*p* < 0.05; **—*p* < 0.01; ***—*p* < 0.001.

(1) $$y_{ijkl}\;=\;\mu \;+O_{i}\;+\;M_{j}(O_{i})\;+\;G_{k}\;+\;e_{ijkl}$$

*y*—response variable; *O_i_*—*i*-paternal genetic effect; *M_j_(O_i_)*—*j*-maternal genetic effects nested into *i*—paternal genetic effect; *G_k_*—effect of diet type; and *e*—the error term.

Mean values, standard deviations (SD), standard error of the mean (SEM) and bar plots were made in GraphPad Prism 7.04 (GraphPad Software, San Diego, CA, USA).

## 3. Results

### 3.1. Total Fat Content in Diets

**Figure 1:**
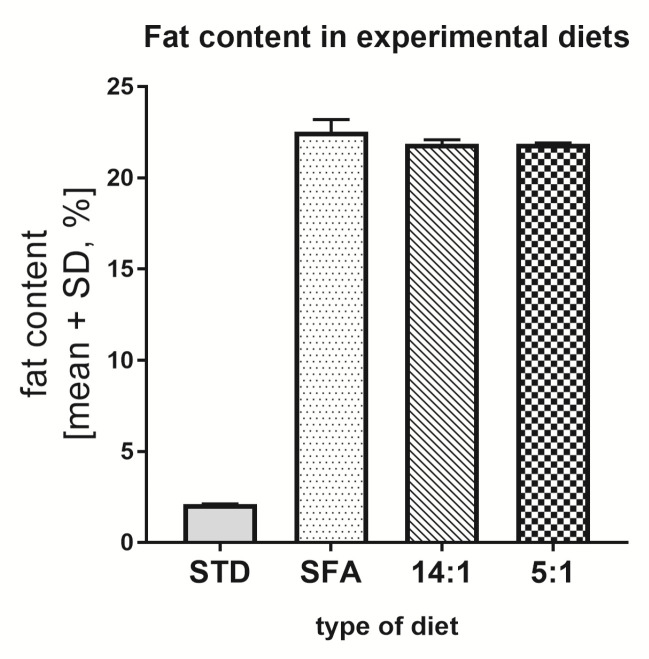
Amount of total fat content in the experimental diets.

The total fat content in the STD was about 2.11%. Whereas the SFA and 14:1 and 5:1 diets contained 22.54%, 21.56% and 21.86% fat, respectively (Figure 1). A more than 10 times higher fat content in the SFA, 14:1 and 5:1 diets than in the STD diet resulted from the addition of vegetable oils.

**Figure 2:**
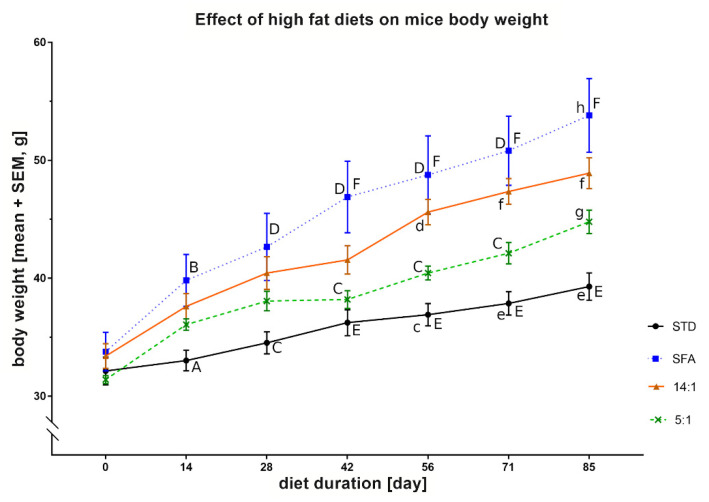
Body weights of mice fed standard (STD), high-fat high-saturated (SFA) diets and high-fat high-polyunsaturated diets with LA/ALA equal 14:1 and 5:1 (14:1 and 5:1, respectively) during approximately 3 months of the experiment. Values are means ± SEM, n = 8. Values with a different letter differ significantly at the same diet duration time point: ^AB^—*p* < 0.05; ^CD^ or ^cd^—*p* < 0.01; ^EF^ or ^ef^ or ^gh^—*p* < 0.001.

Body weights of mice initially did not differ. Body weights of the STD and SFA groups varied after 14 days (^AB^—*p* < 0.05), and mice from the SFA group were significantly heavier than corresponding animals from the STD group after 28 and 42 days of the experiment (^CD^—*p* < 0.01 and ^EF^—*p* < 0.001, respectively). The latter trend continued until the end of diet treatment. After 42 days of the SFA diet, the mice weighed more than the 5:1 group (^CD^—*p* < 0.01), and they gained proportionally more weight after 85 days (^gh^—*p* < 0.001). Body weights in the 14:1 group after 56 days of the experiment were greater than in the STD group (^cd^—*p* < 0.01). Similarly, after another two weeks, body weights of mice from the 14:1 group were higher than the STD group (^ef^—*p* < 0.001). This trend was maintained after 85 days of feeding with respective diets (Figure 2).

### 3.2. Biochemical Blood Parameters

Changes in blood plasma parameters in mice fed the STD diet, SFA diet, 14:1 diet and 5:1 diet for three months were presented in Table 2. There were no significant statistical differences between the experimental groups in any of the parameters measured except for total cholesterol and glucose concentrations. The total cholesterol levels in mice fed the SFA and 14:1 diets were significantly higher than in animals fed the STD diet. Moreover, high-fat diets (SFA, 14:1, and 5:1 diets) caused a significant increase in glucose concentration compared to the STD diet.

### 3.3. Analysis of Liver Proteome Differences

**Figure 3:**
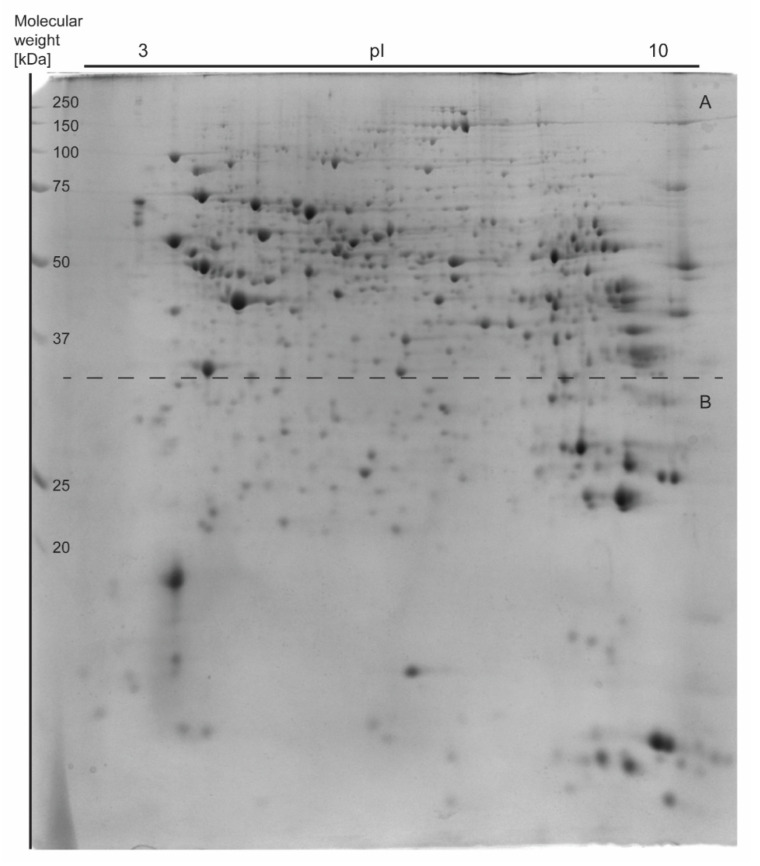
The representative two-dimensional electrophoresis gel image of mouse liver proteins. A total of 600 μg of whole liver proteins was applied to the IPG strip (non-linear pH 3–10, 17-cm length) for the first dimension and then the second dimension was performed on 12% SDS-PAGE gels. The 2DE gel (18 cm × 20 cm × 1.00 mm) was stained with Coomassie Brilliant Blue G-250. The figure was divided into two parts (A and B) according to the dashed line.

**Figure 4:**
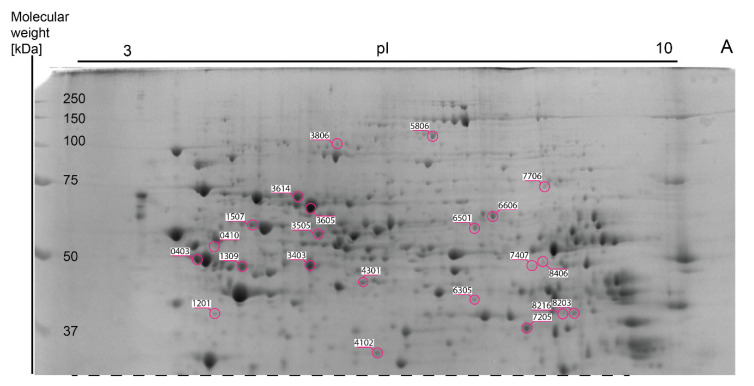
Part A of Figure 3 eresentative 2DE map of liver proteins stained with Coomassie Brilliant Blue G-250). Spot numbers (SSP) and areas of differential spots in the figure correspond to the SSP number in Table 3.

**Figure 5:**
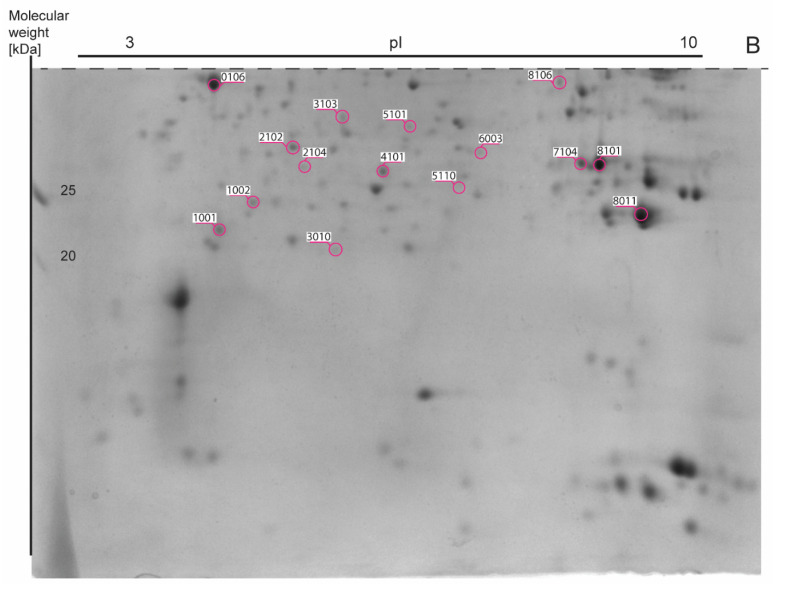
Part B of Figure 3 (representative 2DE map of liver proteins stained with Coomassie Brilliant Blue G-250). Spot numbers (SSP) and areas of differential spots in the figure correspond to the SSP number in Table 3.

Approximately 977 spots were recorded in one 2DE gel with 510 spots overlapping in each of 64 2DE gels. The mean coefficient of variation (CV) of the STD group was 46.47%, 42.04% in the SFA group, 43.29% in the 14:1 group and 42.83% in the 5:1 group. Thirty-seven gel spots showed a significant change in abundance. The results are shown in representative images in Figure 3, Figure 4 and Figure 5.

The list of gel spots whose densitometric intensity varied between the groups and the results of MALDI-TOF protein identification associated with each spot are presented in Table 3. Comparative mass spectrometric analysis of mouse liver extracts identified a total of 35 proteins. Mitochondrial ATP synthase subunit beta (ATP5F1B) and carbonic anhydrase 3 (CA3) were identified in two spots. MASCOT scores of the protein assignments ranged from 61 to 346 (average MASCOT score—161). Proteins were identified with an average of 46% sequence coverage based on 5 to 32 matching peptides. The median number of peptides matches was 13. Median E-values ranged from 1.70 × 10^−30^ to 4.90 × 10^−2^.

The altered liver proteins were grouped into functional sets based on the STRING database, which was presented with a green–white–red fold of change value scale.

**Figure 6:**
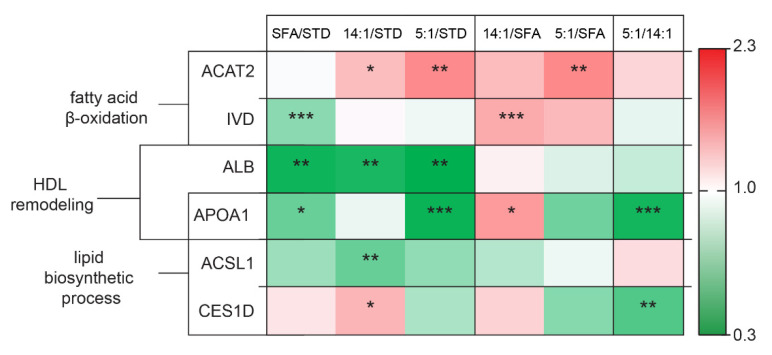
Changes in the abundance of proteins involved in lipid metabolism. The statistically significant (*p* < 0.05) value of the average intensity of the SFA, 14:1 and 5:1 groups compared to the STD group; the 14:1 and 5:1 groups compared to the SFA group; the 5:1 group compared to the 14:1 group. The significance of differences estimated using the model (1). *—*p* < 0.05; **—*p* < 0.01; ***—*p* < 0.001.

Changes in the expression of hepatic proteins involved in lipid metabolism were presented in Figure 6. Significantly higher values of protein abundance were obtained for fatty acid β-oxidation proteins—ACAT2 in the 5:1 group compared to the SFA group (*p* < 0.01) and IVD in the 14:1 group relative to the SFA group (*p* < 0.001). Moreover, ACAT2 intensity was increased in the 14:1 and 5:1 groups compared to the STD group (*p* < 0.05 and *p* < 0.01, respectively). There was a significantly lower protein abundance of IVD in the SFA group than in the STD group (*p* < 0.001). Significantly lower values (more than 2-fold lower) were obtained for albumin after high-fat diets (SFA, 14:1 and 5:1) in comparison to the STD group (*p* < 0.01). The mean values for APOA1 abundance were lower for the SFA and 5:1 diets in comparison to the STD group (*p* < 0.05 and *p* < 0.001, respectively). Statistically significant differences in APOA1 abundance were also found between the 5:1 and 14:1 groups (*p* < 0.001). Expression of other proteins involved in lipid biosynthesis was also decreased, inter alia ACSL1, whose expression was reduced in the 14:1 compared to the STD group, and CES1D, whose expression was lower in the 5:1 versus the 14:1 group (*p* < 0.01).

**Figure 7:**
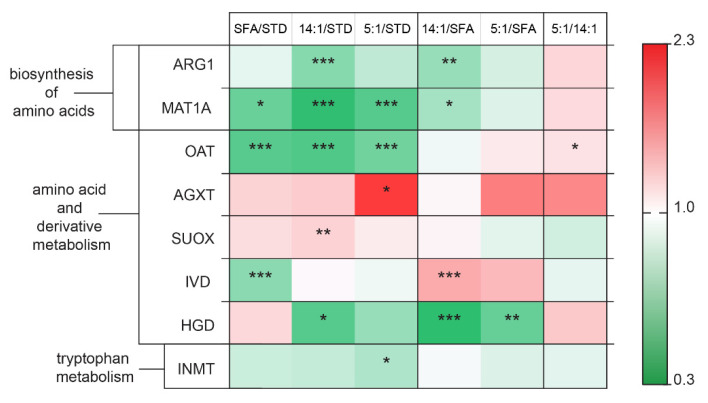
Changes in the abundance of proteins involved in amino acid metabolism. The statistically significant (*p* < 0.05) value of the average intensity of the SFA, 14:1 and 5:1 groups compared to the STD group; the 14:1 and 5:1 groups compared to the SFA group; the 5:1 group compared to the 14:1 group. The significance of differences estimated using the model (1). *—*p* < 0.05; **—*p* < 0.01; ***—*p* < 0.001.

Diets enriched with PUFAs or SFAs affected hepatic proteins involved in amino acid metabolism (Figure 7). In the 14:1 group, the quantity of ARG1 was reduced in comparison to the STD (*p* < 0.001) and SFA groups (*p* < 0.01). In the SFA, 14:1, and 5:1 groups, reductions were observed in the level of MAT1A (*p* < 0.05, *p* < 0.001, *p* < 0.001, respectively) and OAT (all—*p* < 0.001) in comparison to the STD group. After three months of diet, AGXT levels significantly increased in the 5:1 group compared to the STD group (*p* < 0.05). The level of SUOX was increased in the 14:1 group compared to the STD group (*p* < 0.01). Moreover, increased abundance of IVD was determined in the 14:1 group compared to the SFA group (*p* < 0.001); similarly lower levels of HGD were also found in the 14:1 and 5:1 groups versus the SFA group (*p* < 0.001 and *p* < 0.01, respectively). There were significant reductions in the abundance of IVD, HGD and INMT in the SFA, 14:1 and 5:1 groups, respectively, in comparison to the STD group (*p* < 0.001, *p* < 0.05 and *p* < 0.05, respectively).

**Figure 8:**
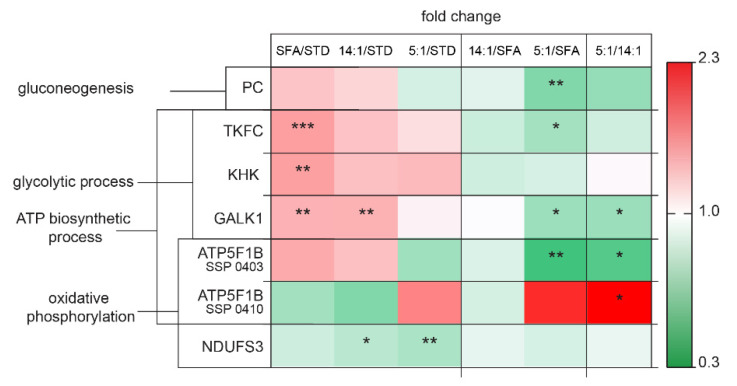
Changes in the abundance of proteins involved in carbohydrate metabolism and ATP synthesis. The statistically significant (*p* < 0.05) value of the average intensity of the SFA, 14:1 and 5:1 groups compared to the STD group; the 14:1 and 5:1 groups compared to the SFA group; the 5:1 group compared to the 14:1 group. The significance of differences estimated using the model (1). *—*p* < 0.05; **—*p* < 0.01; ***—*p* < 0.001.

Liver proteome analyses showed an influence of the treatment with different diets enriched with PUFAs or SFAs on several proteins responsible for carbohydrate metabolism and ATP synthesis (Figure 8). The level of hepatic PC decreased successively in the 5:1 group in comparison to the SFA group (*p* < 0.01). Levels of proteins involved in glycolytic processes, such as TKFC, KHK and GALK1, were all significantly increased in the SFA group in relation to the STD group (*p* < 0.01). GALK1 level was also increased in the 14:1 group compared to the STD group (*p* < 0.01). In contrast, TKFC and GALK1 expression was decreased in the 5:1 compared to the SFA group (*p* < 0.05). Additionally, GALK1 abundance was also decreased in the LA/ALA 5:1 group compared to the 14:1 group (*p* < 0.05). Two isoforms of ATP5F1B, including SSP 0403 and SSP 0410, showed differentiated changes in mouse livers, ATP5F1B SSP 0403 from the 5:1 group was decreased compared to the 14:1 group (*p* < 0.05), while expression level of ATP5F1B SSP 0410 in the 5:1 group was more highly expressed compared to the 14:1 group (*p* < 0.05). Additionally, ATP5F1B SSP 0403 expression was shown to be reduced in the 5:1 group versus the SFA group (*p* < 0.01).

**Figure 9:**
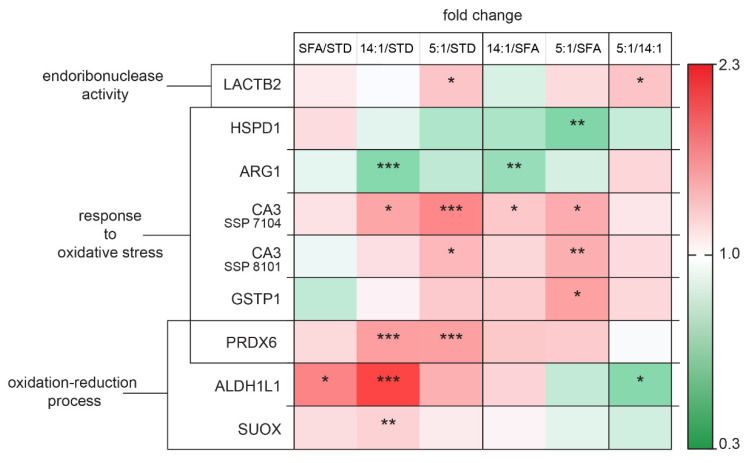
Changes in the abundance of proteins involved in oxidative stress regulation. The statistically significant (*p* < 0.05) value of the average intensity of the SFA, 14:1 and 5:1 groups compared to the STD group; the 14:1 and 5:1 groups compared to the SFA group; the 5:1 group compared to the 14:1 group. The significance of differences estimated using the model (1). *—*p* < 0.05; **—*p* < 0.01; ***—*p* < 0.001.

The heat map in Figure 9 demonstrates the clustering of significantly increased versus significantly decreased expression of proteins involved in oxidative stress in liver tissue in mice fed with high-polyunsaturated fatty acid diets compared to tissues of mice fed with the standard diet or high-saturated fatty acid diet. Endoribonuclease LACTB2 levels were markedly elevated in the 5:1 samples in comparison to the STD and 14:1 samples (*p* < 0.05). The expression of HSPD1 was down-regulated in the 5:1 group compared to the SFA group (*p* < 0.01). ARG1 was identified as a down-regulated protein in the 14:1 group relative to the STD and SFA groups (*p* < 0.001 and *p* < 0.01, respectively). Two up-regulated proteins found in the 5:1 group in comparison to the SFA group included CA3 and GSTP1. High levels of PRDX6 were found in the liver of mice fed the 14:1 and 5:1 diets compared to the STD group (*p* < 0.001).

**Figure 10:**
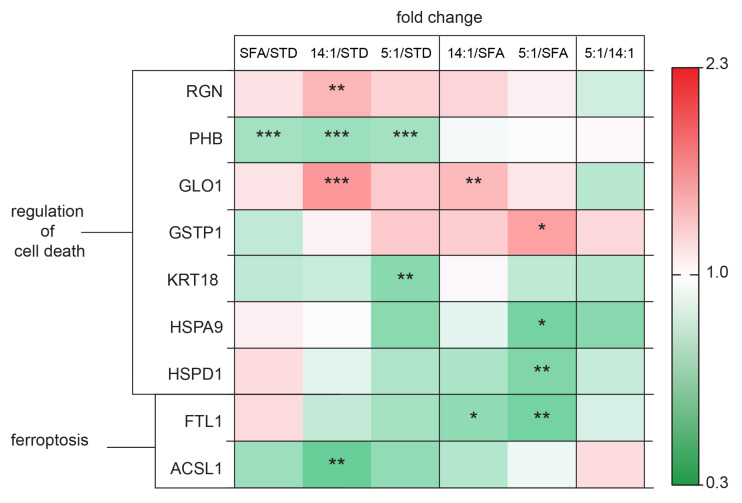
Changes in proteins involved in cell death processes. The statistically significant (*p* < 0.05) value of the average intensity of the SFA, 14:1 and 5:1 groups compared to the STD group; the 14:1 and 5:1 groups compared to the SFA group; the 5:1 group compared to the 14:1 group. The significance of differences estimated using the model (1). *—*p* < 0.05; **—*p* < 0.01; ***—*p* < 0.001.

Our results of proteomic screening provided a list of proteins involved in cell death processes (Figure 10). RGN and GLO1 levels were increased in the 14:1 group in comparison to the STD group (*p* < 0.01 and *p* < 0.001, respectively). The animals fed the 14:1 diet had significantly increased GLO1 level compared to the SFA group (*p* < 0.01). In contrast, GSTP1 expression was markedly decreased in the 5:1 group compared to the SFA group (*p* < 0.05).

PHB levels were decreased following the HFD treatment (SFA/STD, 14:1/STD and 5:1/STD, *p* < 0.001). KRT18 protein expression changed significantly following 3 months of feeding the PUFA diet (LA/ALA ratio—5:1) in comparison to the STD group (*p* < 0.01). The levels of HSPA9 and HSPD1 proteins in the 5:1 group were significantly lower in relation to the SFA group (*p* < 0.05 and *p* < 0.01, respectively). FTL1 expression was decreased in the groups of mice fed the high-PUFA diets compared to the group fed the high-saturated diet (14:1/SFA *p* < 0.05 and 5:1/SFA *p* < 0.01, respectively). We observed a lower level of ACSL1 in mice treated with the 14:1 diet compared to the group fed the standard diet (*p* < 0.01).

After three months of the STD diet, the expression of 13 proteins was different in comparison to the SFA group, including elevated levels of six proteins: ALB (SSP 3605), APOA1 (SSP 1002), IVD (SSP 6305), MAT1A (SSP 3403), OAT (SSP 4301) and PHB (SSP 2102) as well as reduced levels of seven proteins: ALDH1L1 (SSP 3806), GALK1 (SSP 1201), GPD1 (SSP 8106), HMGCS2 (SSP7407), KHK (SSP 3103), TKFC (SSP 6606) and UniProtKB: Q91V76 (SSP 4102).

The differences in the expression of 11 protein between the 14:1 and SFA diets were identified, involving higher levels of the following five proteins: APOA1 (SSP 1002), GLO1 (SSP 1001), HDHD3 (SSP 6003), IVD (SSP 6305) and CA3 (SSP 7104). Expression downregulation was observed the case of six proteins in the 14:1 group in comparison to the SFA group: ARG1 (SSP 7205), FTL1 (SSP 3010), GPD1 (SSP 8106), HGD (SSP 8406), HMGCS2 (SSP 7407) and MAT1A (SSP 3403).

The level of eleven proteins was altered in the 5:1 group compared to the SFA group. The expression of three proteins was elevated in the 5:1 group: ACAT2 (SSP 8203), CA3 (SSP 7104 and SSP 8101) and GSTP1 (SSP 8011). The level of eight proteins in the 5:1 group was decreased in comparison to the SFA group: ATP5F1B (SSP 403), GALK1 (SSP 1201), HSPD1 (SSP 1507), FTL1 (SSP 3010), HSPA9 (SSP 3614), PC (SSP 5806), TKFC (SSP 6606) and HGD (SSP 8406).

After three months of feeding the high-PUFA diets (14:1 and 5:1) the level of CA3 was increased in comparison to the SFA group. Two protein spots—FTL1 and HGD—were identified that were consistently reduced in the liver proteome of mice fed high PUFA diets in comparison to the SFA diet.

The present proteomic study also revealed differences between groups fed high-PUFA diets in the levels of hepatic proteins, including downregulated proteins in the 5:1 group (ALDH1L1, APOA1, CES1D, GALK1) and three upregulated proteins in the 5:1 group (GPD1, LACTB2, OAT) in comparison to the 14:1 group.

**Figure 11:**
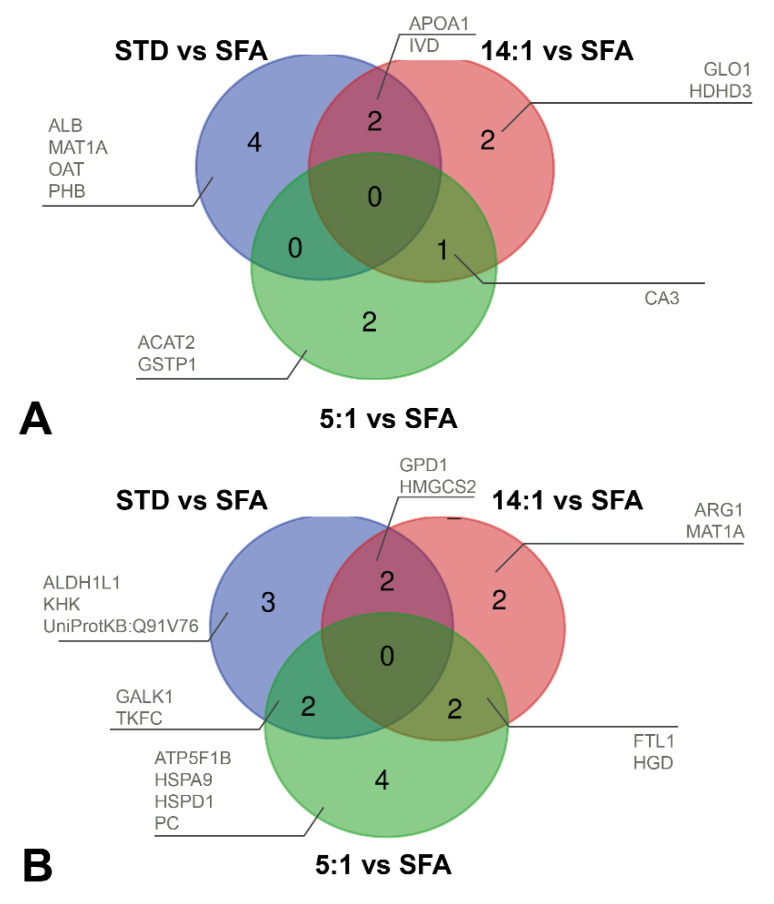
Venn diagram of differentially expressed proteins after three-months diets. (**A**) The number and list of proteins with increased expression in the STD (blue), 14:1 (red) and 5:1 (green) groups compared to the SFA. (**B**) The number and list of proteins with decreased expression in the STD (blue), 14:1 (red) and 5:1 (green) groups compared to the SFA.

A detailed comparison of increased and decreased protein expressions between experimental groups is presented in Figure 11A,B.

### 3.4. Controlling mRNA and Protein Expression Levels by High-Fat Diets

OAT and PRDX6 were further analyzed as representatives of amino acid metabolism and oxidative stress regulation processes, respectively. The mRNA and protein expression levels of these proteins were measured using real-time PCR and immunoblotting. The specificity of primary antibodies and primers were confirmed. Only one amplification product was detected for each of the primer pairs. The PCR reaction specificity was ensured by post-amplification melting-curve analysis. Uncropped Western blots, amplicon products and melting curves were available in the Supplementary Materials (Figures S1–S3).

**Figure 12:**
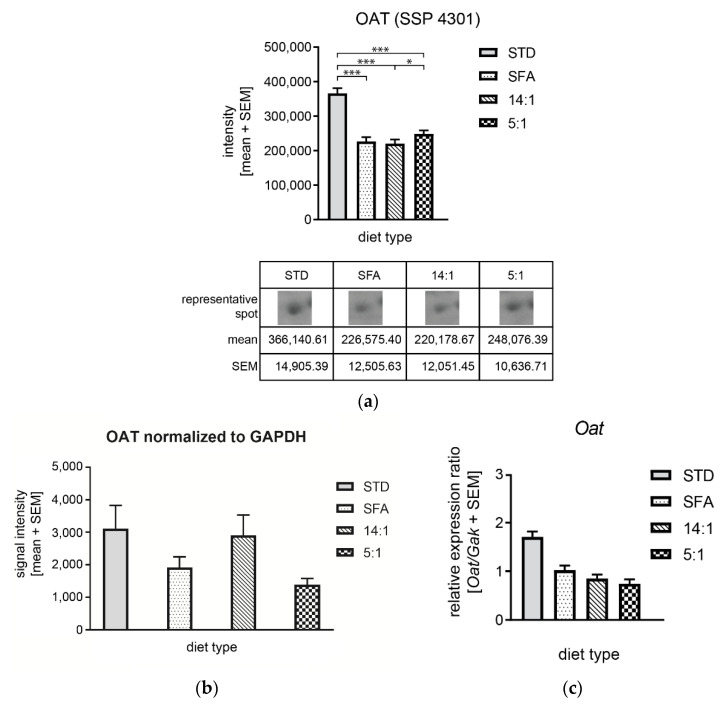
Alterations of OAT expression in response to different high-fat diets. (**a**) Protein levels based on 2DE; (**b**) Western blotting analysis using anti-OAT and anti-GAPDH antibodies and six mouse liver samples per group; signal intensities of OAT were normalized to GAPDH signal intensities. Graphs presented a mean intensity with a standard error of the mean (SEM); (**c**) *Oat* mRNA level normalized to *Gak.* *—*p* < 0.05; ***—*p* < 0.001.

**Figure 13:**
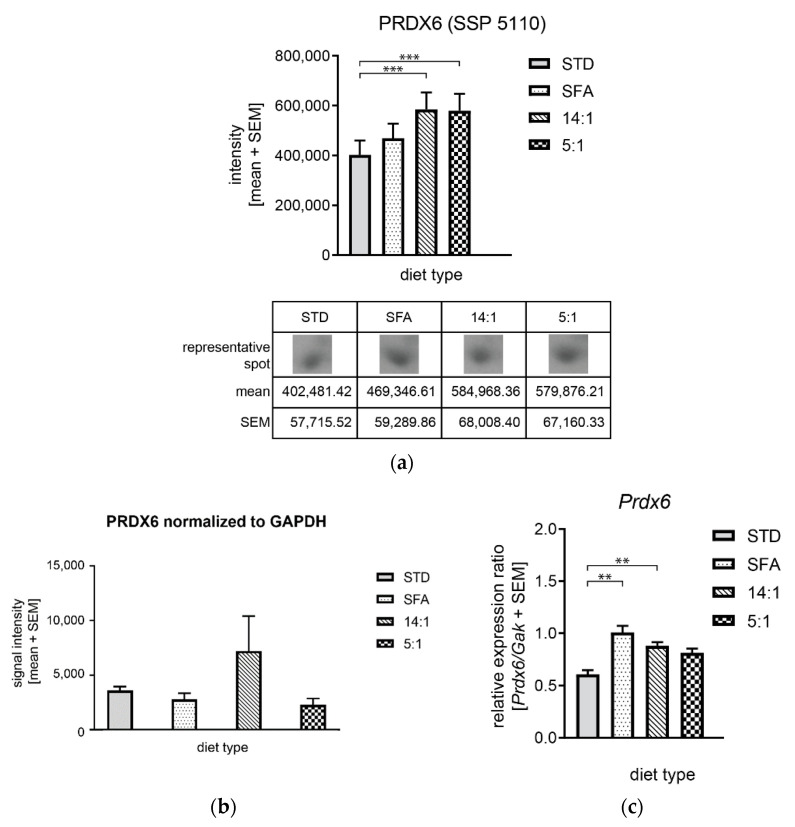
Alterations of PRDX6 expression in response to different high-fat diets. (**a**) Protein levels based on 2DE; (**b**) Western blotting analysis using anti-PRDX6 and anti-GAPDH antibodies and six mouse liver samples per group; signal intensities of PRDX6 were normalized to GAPDH signal intensities. Graphs presented a mean intensity with a standard error of the mean (SEM); (**c**) *Prdx6* mRNA level normalized to *Gak.* **—*p* < 0.01; ***—*p* < 0.001.

No significant differences were observed in OAT and PRDX6 protein levels (Figure 12a and Figure 13a). As shown in Figure 12b, OAT expression was insignificantly reduced in the high-fat diet groups (SFA and 5:1) in comparison to the STD group, but the reduction pattern was consistent with 2DE data. The expression of OAT gene at the mRNA level was not significantly increased in animals fed with the STD diet compared to the other experimental groups (Figure 12c).

Real-time PCR results were consistent with the 2DE results showing the increased PRDX6 mRNA level in the 14:1 group compared to the STD group, while increased *Prdx6* expression in the 5:1 group was not confirmed. The immunoblot analysis did not confirm changes in PRDX6 levels between groups (Figure 13b).

## 4. Discussion

The study presented the influence of HFDs with different fatty acid composition on the mouse liver proteome. In the experiment, we assigned two control groups. Animals of the control group (STD) received standard chow for laboratory mice. The SFA group was used as a negative control in comparisons between high-fat diet groups, according to a high content of saturated fatty acids with a chain length of 12–18 carbons and a low PUFA content in coconut oil. We compared the effects of a high LA/ALA ratio (14:1) with the effects induced by a lower ratio (5:1) to investigate the impact of high-fat diets with different n–6/n–3 polyunsaturated fatty acids contents on liver metabolism. These two LA/ALA proportions mimic the high ratio of these fatty acids (14:1) specific to a Western-type diet, and the low ratio of these acids (5:1) is considered a healthy diet.

It was shown that the composition of consumed fat and the content of individual fatty acids had a different effect on weight gain [31]. In the present study, we noticed differences in weight gain between the experimental groups, as body weight gain was higher in the SFA group in comparison to the STD group after the first two weeks of the experiment. Body weights in the SFA group were significantly increased when compared to the STD group and the group fed the high-fat diet with the recommended n–6/n–3 (5:1) ratio. These results were consistent with the study of Beulen et al. who showed that replacing SFAs with PUFAs in the diet resulted in body weight loss [32]. One possible explanation could be a different fat accumulation, as shown in the studies by Rosqvist et al. where the group consuming SFAs was characterized by an increased fat accumulation in the liver and viscera compared to the group ingesting more PUFAs [33]. Increased uptake of this feed due to its attractive aroma, which was caused by a characteristic flavor of virgin coconut oil, could be another factor affecting body weight of the SFA mouse group. In addition, after 56 days of the diet, body weights of mice from the 14:1 group were elevated compared to the STD group, and this difference increased during the following four weeks of the experiment. However, the current study showed that the SFA diet was more obesogenic than the 14:1 and 5:1 PUFA diets.

In this study, high-fat diets (SFA and 14:1) increased total cholesterol levels compared to the standard low-fat diet, which was consistent with the results of Li et al. who reported that total plasma cholesterol levels were significantly higher in mice fed a high-fat diet after 12 and 16 weeks of the experiment compared to a low-fat diet group [34,35]. However, supplementation with a high-fat diet enriched in n–3 acids (5:1 diet) did not significantly change total cholesterol content in comparison to a standard diet. This could indicate that a high-saturated fat diet and a diet high in n–6 FAs disturbed cholesterol homeostasis to a higher extent than the high-PUFA 5:1 diet. This was partially in line with the findings of Kralova Lesna et al. who confirmed that PUFA diet consumption resulted in a substantially lower concentration of total cholesterol than in the SFA diet [36]. A similar result was also obtained by Gaundal et al. as these authors demonstrated that replacing products rich in SFAs with high amounts of PUFAs for only three days in young but adult men and women, resulted in a reduction of total cholesterol levels in blood serum [37].

We also found that feeding HFDs rich in PUFAs for three months did not change blood plasma biochemical parameters, including albumin, triglycerides, LDL and HDL lipoproteins. This observation differed slightly from the study by Diniz et al. who reported that albumin and HDL cholesterol levels were higher in PUFA-fed rats than in SFA-fed rats, however triacylglycerol and LDL cholesterol levels were lower after the administration of PUFA diets compared to the SFA diet [38]. These differences could be due to the shorter treatment period (5 weeks in the study of Diniz vs. 12 weeks in the present study).

### 4.1. Differences in the Expression of Proteins Related to Lipid Metabolism

Soltis et al. showed that a high-fat diet caused liver dysfunction, including abnormal hepatocyte morphology and apoptosis rate [39]. Liver steatosis may lead to its dysfunction, inflammation, fibrosis, and consequently, to cirrhosis and hepatocellular carcinoma. Previous studies demonstrated that fat accumulation in the liver caused by a high-fat diet was associated with increased lipogenesis and inhibition of lipolysis [40]. Similar results were not obtained in the present study. The expression of proteins related to lipid biosynthesis processes, such as APOA1 and ACSL, was reduced following the high-fat diet. Moreover, none of the altered proteins was related to lipolysis.

Our results clearly showed that all high-fat diets reduced ALB protein level compared to the STD diet. Additionally, concentrations of two other proteins related to lipid metabolism—ACAT and IVD—were increased after PUFA-rich diets compared to the SFA diet. We also identified proteins whose expression was altered between PUFA-rich diets: APOA1 and CES1D levels were decreased in the 5:1 group compared to the 14:1 group.

Proteomic studies performed here indicated that different types of high-fat diets also altered the abundance of proteins associated with lipid metabolism, such as ACAT2. This protein is mainly expressed in the liver, but also in the small intestine [41]. ACAT2 is an endoplasmic reticulum transmembrane protein that is responsible for transferring the fatty acyl moiety of acyl-CoA to free cholesterol by producing cholesteryl esters (CE), mainly cholesteryl oleate and palmitate. ACAT2-derived CE can be packaged directly into apoB-containing lipoproteins or stored as neutral lipid droplets in the cytosol [42]. A significantly higher abundance of ACAT2 protein was observed in the 14:1 and 5:1 groups than in the control group, and in the 5:1 group compared to the SFA group. This result has confirmed that PUFAs are preferred fatty acid substrates for ACAT2 in the liver and may stimulate ACAT activity as opposed to SFAs. The consequence of increased hepatic ACAT2 activity was a higher secretion of cholesteryl ester from the liver and subsequent enrichment of LDL with cholesteryl oleate [43]. Bell et al. fed ACAT2^−/−^ mice with diets enriched in either n–3 or n–6 PUFAs, saturated FAs and *cis* or *trans* monounsaturated FAs. After 20 weeks of the diet, the control group showed signs of atherosclerosis signs, whereas ACAT2^−/−^ mice were protected against atherosclerosis regardless of the administered fat [43]. On the other hand, ACAT2^–/–^ mice were shown to be more susceptible to insulin resistance caused by a high-fat diet [41]. It is interesting because animals fed PUFA-rich HFDs with the highest expression of ACAT2, simultaneously showed slightly lower plasma glucose level. Moreover, ACAT2 expression was shown to be stabilized by saturated fatty acids and sterols through the ROS formation, which prevented its degradation via ubiquitin-proteasome pathway [41]. It has indicated that different expression pattern may be observed for this protein depending on its particular modifications.

IVD was another protein associated with lipid metabolism differentially expressed in response to 3 months feeding with high-fat diets. This protein level was higher in the 14:1 than in the SFA group (*p* < 0.001). Additionally, IVD expression was decreased in the SFA group compared to the STD group (*p* < 0.001). A contrasting result was obtained by Guo et al. who showed that mice fed a high fat died had elevated IVD levels in the liver compared to mice fed a normal diet, which was suggested as enhanced branched-chain amino acid (BCAA) degradation in liver mitochondria of mice fed the high-fat diet [44]. It should be emphasized, however, that IVD downregulation may lead to the accumulation of isovaleryl-CoA derivatives, including isovaleric acid (IVA), and reduced production of acetyl-CoA and acetoacetate [45]. The consequences of IVA accumulation may be the induction of oxidative stress and protein oxidative damage due to significantly elevated carbonyl formation [46]. This suggested that a marked downregulation of IVD could induce oxidative stress in the livers of animals fed the SFA diet.

APOA1 is the main protein forming HDL particles. The highest HDL concentration in blood plasma was recorded in the 14:1 group. Chylomicrons enriched in n–6 PUFAs, such as linoleic acid, are enzymatically removed from circulation faster than chylomicrons enriched in SFAs or n–3 PUFAs [47]. Reduced fat mass was observed in mice overexpressing APOA1 [48]. Similarly, ApoA-I^−/−^ mice showed increased body weight compared to wild type mice fed an obesogenic diet [49]. In this study, increased APOA1 levels in the liver of 14:1 mice, compared to the SFA group (*p* < 0.05), could be a reason for lower weight gains by 14:1 mice compared to SFA mice. It may also be the reason why 14:1 mice exhibited increased circulating cholesterol levels compared to the SFA and 5:1 groups (but results were not significant). Han et al. reported that Pepck and G6Pase mRNA levels were significantly elevated in the livers of Apoa-I-deficient mice (ApoA-I^−/−^). Plasma glucose concentrations in the latter animals were significantly increased compared to wild-type controls. This suggests that APOA-I decrease can modulate critical gluconeogenic enzymes [50]. This observation was consistent with our results, where the level of APOA1 protein was lower in the livers of mice from the SFA and 5:1 groups compared to the STD group, and could also be the reason for increased plasma glucose levels. An in-depth analysis of the detected change in the expression of apolipoprotein A1 at the protein level under the influence of diets with a different LA to ALA ratio (*p* < 0.001) should be the direction of further research.

CES1D was another identified protein whose expression differed between mice fed different LA/ALA diets, as it was decreased in the 5:1 group versus the 14:1 group (*p* < 0.01); murine CES1D is also annotated as triacylglycerol hydrolase (TGH) or CES3. Lian et al. showed that CES1D deficiency attenuated both simple hepatic steatosis and irreversible NASH [51]. Mice deficient in CES1D expression ameliorated high-fat diet-induced hepatic steatosis by reducing of de novo lipogenesis [51]. In addition, hepatic CES1D is involved in the supply of substrates for VLDL assembly. CES1D inhibition decreased VLDL secretion both in vitro and in vivo [51]. Moreover, fatty acid oxidation was also significantly increased in hepatocytes isolated from Ces1d-deficient mice [52]. VLDL level and steatosis and fibrosis markers were not determined in the present study, but a high-PUFA diet with 5:1 LA/ALA ratio has the potential to alleviate fatty liver disease and prevent steatohepatitis in comparison to a high-PUFA diet with LA/ALA of 14:1.

### 4.2. Changes in the Expression of Proteins Involved in Carbohydrate Metabolism

Our study showed increased blood glucose levels after 12-week feeding of all types of high-fat diets compared to the standard diet; the highest glucose level was recorded after the SFA diet treatment. Our results were consistent with the work of He et al. [34]. Almost all circulating glucose is derived from hepatic glycogenolysis (breakdown of stored glycogen) and de novo production of glucose from precursors such as pyruvate and galactose (i.e., gluconeogenesis, which is stimulated by glucagon and inhibited by insulin) [53].

The present study showed that the liver proteome after three months of the SFA diet was characterized by an increased level of TKFC, KHK and GALK1 proteins in comparison to the STD diet. These proteins are involved in the glycolytic process. Hepatic fructose metabolism, represented by two enzymes (KHK and TKFC) was changed following the high-fat-high-saturated diet (SFA group) in comparison to the standard diet (STD group). Nevertheless, these alterations did not occur after high-fat-high-PUFA diets in contrast to the STD group. KHK, the first enzyme of fructose metabolism, provides substrates for fatty-acid synthesis and enhances de novo lipogenesis. In this way, the SFA diet could lead to fatty liver disease and insulin resistance pathogenesis, as elevated KHK protein levels were found in liver biopsies of obese adolescent humans with NAFLD [54]. Alterations in the second step of hepatic fructolysis were also observed in the form of changed TKFC protein levels. The SFA diet significantly increased the level of TKFC protein compared to the STD group (*p* < 0.001). However, TKFC expression in the 5:1 group was reduced compared to the SFA group (*p* < 0.05).

We found a set of 3 proteins involved in glucose metabolism that were down-regulated in mice liver by the high-PUFA 5:1 diet compared to the SFA diet. This category included proteins involved in gluconeogenesis (PC, *p* < 0.01) and glycolysis (GALK1 and TKFC, *p* < 0.05). Increased hepatic gluconeogenesis is a critical step in the pathogenesis of type 2 diabetes. PC protein regulates hepatic glucose production. Chronically decreased PC expression in liver and adipose tissue was demonstrated to reduce plasma glucose concentrations, the rate of endogenous glucose production, adiposity, plasma lipid concentrations, hepatic steatosis and improved hepatic insulin sensitivity [55]. Thus, the 5:1 diet may be more therapeutic in reducing fasting hyperglycemia in humans with type 2 diabetes than the 14:1 and SFA diets. Additionally, two high-fat diets (SFA and 14:1) increased GALK1 levels in the liver in comparison to the STD group (*p* < 0.01). The human body metabolizes galactose through the Leloir pathway, and in the first step, GALK, which phosphorylates α-D-galactose to galactose-1-phosphate (gal-1-P), is the rate-limiting enzyme in the clearance of galactose from the blood [56,57]. Increased expression of GALK may result in the accumulation of toxic galactose-1-phosphate. On the other hand, the SFA and 14:1 diets may be beneficial in the management of galactokinase deficiency (galactosemia type II) to avoid blocking the Leloir pathway leading to galactitol accumulation in the lens [58].

### 4.3. Changes in the Expression of Proteins Associated with Amino Acid Metabolism.

The present proteomic study revealed alterations between the levels of proteins involved in amino acid metabolism in the liver, as a result of different types of high-fat diet digestion. The expression of MAT1A and OAT was decreased in all experimental high-fat groups compared to the STD group. There were also proteomic alterations in ARG1, HGD and MAT1 hepatic levels between the high-PUFA 14:1 group and SFA group. Moreover, one protein was upregulated in the group of mice fed a diet with a 5:1 n–6/n–3 PUFA ratio compared to mice fed a diet with a 14:1 ratio.

ARG1 expression was decreased in the liver of mice fed the 14:1 diet in comparison to mice fed the SFA diet (*p* < 0.01). A downregulation of ARG1 expression was observed in the 14:1 group as opposed to the STD group (*p* < 0.001). Similar results were obtained in the study of Bashir et al. where ARG1 protein and mRNA levels were reduced in murine macrophages in adipose tissue of the group fed an HFD in comparison to the lean group fed standard chow [59]. L-Arginine (Arg), hydrolyzed by arginase, is considered an important amino acid in homeostasis maintenance, and its metabolism is altered in various diseases [60]. Arginase I (ARG1) is the most abundantly expressed isoform in hepatocytes. Furthermore, liver damage was shown to increase plasma arginase activity in obese rodents [61,62]. A study by Romero et al. in diabetic rats revealed significantly increased arginase I levels in the aorta and liver, as well as elevated vascular and hepatic arginase activity [63]. A diet rich in PUFA with LA/ALA of 14:1 has been found to be an effective therapeutic strategy to reduce the level of this protein. In addition, this is because arginase inhibition may be a treatment option for obesity-induced vascular endothelial dysfunction, hepatic lipid abnormalities, whole-body adiposity and obesity-related hypertension [61,64,65].

The current work showed a reduced level of OAT protein in the groups fed high-fat diets compared to the group fed the STD diet. A reduction in OAT expression at the protein level was observed in the livers of mice fed with PUFA-rich feed (14:1 and 5:1) compared to mice fed with SFAs. There were also differences in the level of this protein between the groups fed with different PUFA proportions (14:1 and 5:1). Animals fed the 5:1 diet showed higher OAT gene expression at the protein level. This result was consistent with similar analyses using two-dimensional electrophoresis, in which a diet rich in PUFA n–3 with a lower n–6/n–3 ratio (5:1) increased the level of OAT expression and its activity in the liver compared to animals receiving a diet with an n–6/n–3 ratio of 30:1 [66]. Our results differed from those obtained by Luo et al. [67], as the latter authors demonstrated increased expression of the *Oat* gene at the protein level in the livers of mice fed a high-fat diet compared to the group of animals fed a standard-fat diet. However, disparate results between the studies could be due to different total fat contents in the feeds (10% and 60%) used by Luo et al. [67]. OAT is a key enzyme in the urea cycle that metabolizes ornithine to glutamate semialdehyde in the liver. Nevertheless, its overexpression in transgenic mice did not alter amino acid homeostasis [68]. *Oat* is a PPARα-regulated gene [69] and its activation by PUFA induces the expression of genes with the PPRE sequence, including ACOX and CPT-I [70]. High expression of the *Oat* gene is characteristic of hepatocellular carcinoma (HCC); for this reason, it is supposed to be involved in carcinogenesis. The ability of OAT inhibitors to limit HCC growth makes OAT a potential therapeutic target for inhibiting the growth of this tumor [71]. For this reason, higher fatty acid contents in the diet, in particular *n*–6 PUFAs, may inhibit tumorigenesis in the liver and act similarly to synthetic OAT inhibitors.

Twelve-week interventions with the 14:1 and 5:1 high-PUFA diets led to reduced HGD levels compared to the high-saturated fat diet (*p* < 0.001 and *p* < 0.01, respectively). Similar results were obtained by Mendez et al. who reported that fish oil mixture supplementation of a high-fat high-sucrose (HFHS) diet significantly downregulated HGD level in rat liver compared to a control diet [72]. According to these results, dietary fat restriction should be considered to avoid adverse homogentisic acid (HGA) accumulation, for instance in alkaptonuria patients with residual HGD enzymatic activity caused by missense mutations in the *HGD* gene.

### 4.4. Changes in the Expression of Proteins Involved in Oxidative Stress Regulation

Previous research has shown that long-term feeding with a high-fat diet increases oxidative stress [73]. Among the experimental high-fat diets, the 14:1 and 5:1 groups exhibited altered levels of proteins mainly involved in the response to oxidative stress, including PRDX6, when compared to standard dietary fat levels. However, it should be mentioned that these results were only partially validated at the mRNA level and using immunoblotting at the protein level. PUFAs undergo peroxidation after reacting with free radicals and are converted into reactive free radicals, and their chain reaction products display high biological activity [74]. PRDX6 is known as a bifunctional protein with both glutathione peroxidase and phospholipase A2 activity. Oxidant stress is a potent inducer of PRDX6 expression [75] that reduces H_2_O_2_ and phospholipid hydroperoxides. Lack of PRDX6 is associated with pro-inflammatory gene upregulation in the liver [76]. Overexpression of PRDX6 reduced reactive oxygen species levels and improved cell viability [77]. Moreover, PRDX6 has been shown to maintain mitochondria integrity under oxidative stress and protect against insulin resistance and non-alcoholic fatty liver disease induced by a high-fat diet [78]. Thus, increased hepatic PRDX6 expression in animals fed PUFA-rich HFDs may indicate the contribution of PUFAs to liver protection against oxidative stress caused by high-fat diet administration. Consequently, the lack of a protective effect of PRDX6 overexpression may lead to the development of metabolic disorders.

Extensive hepatic proteomic changes induced by feeding PUFAs revealed proteins associated with oxidative stress response, including HSPD1, CA3 and GSTP1. HSPD1/HSp60 was downregulated in the 5:1 group compared to the SFA group (*p* < 0.01). However, no alterations were observed between HFDs and STD. Our data were not consistent with the results obtained by Guo et al. who showed HSP60 upregulation in liver mitochondria isolated from mice fed an HFD compared to a regular diet [44]. Hsp60 forms a large homo-oligomeric protein complex that assists in folding proteins and protein domains [79]. CA3 belongs to a family of enzymes that catalyze the reversible condensation of water and carbon dioxide to carbonic acid, which further dissociates to bicarbonate. Bicarbonate is considered a necessary precursor for fatty acid synthesis and storage. Thus, CA3 is abundant in tissues that synthesize and store lipids, such as liver and adipose tissue [80]. Zimmerman provided information on the role and mechanism of action of CA3 as an antioxidant by assessing S-glutathionylation and irreversible oxidation of two protein-reactive cysteines in the presence of increasing intensity of oxidative stress damage [81]. In the present study, CA3 level was increased in the livers of animals from the 14:1 and 5:1 groups compared to the STD group. Increased CA3 can exert adverse effects because it is involved in hepatic steatosis development induced by a Western diet containing high fructose corn syrup; CA3 inhibition resulted in a decrease in hepatic steatosis and weight gain [82].

### 4.5. Changes in the Expression of Proteins Involved in Cell Death Processes

Another set of altered hepatic proteins has been associated with cell death processes. In the present study, PHB levels were lower in the livers of mice fed all types of high-fat diets compared to the standard-fat diet (*p* < 0.001). Such results indicate that three-month diets rich in fat may initiate liver dysfunctions, because PHB is known to be a highly conserved, ubiquitously expressed protein that participates in diverse processes, including mitochondrial chaperoning activity, growth and apoptosis. PHB exhibits antioxidant properties in the liver [83,84]. Ko et al. demonstrated that knockout mice lacking PHB1 had impaired mitochondrial function, upregulated expression of genes involved in malignant transformation and liver fibrosis, as well as multiple liver lesions between 35–46 weeks of age [85].

Our results also revealed changes in the expression of other proteins related to cell death processes, including RGN, GLO1 and KRT18, whose levels were increased in the PUFA groups compared to the STD group, while HSPA9, HSPD1 and FTL1 levels were decreased in the 5:1 group compared to the SFA group. RGN is known as a calcium-binding protein that plays a significant role in maintaining intracellular calcium signaling and lipid metabolism [86]. A proteomic study by Ahmed et al. found that mice fed a high n–3 PUFA diet showed lower RGN expression compared to mice fed a low *n*–3 PUFA diet [66]. This result was not confirmed in the current experiments, where the RGN level was elevated in mice fed the 14:1 diet in comparison to the STD group (a low n–3-PUFA diet) (*p* < 0.01). RGN overexpression was shown to induce hepatic insulin resistance and hyperlipidemia in rats [87]. Yamaguchi et al. showed increased serum RGN levels, whereas liver mRNA levels decreased after carbon tetrachloride administration, suggesting a role of this protein in cellular response in chronic liver damage [88]. KRT18 levels markedly decreased following 3 months of feeding the PUFA LA/ALA 5:1 diet in comparison to the STD group (0.73-fold change, *p* < 0.01). KRT18 encodes type I intermediate filament chain keratin 18. Ubiquitinated KRT18, KRT8, and sequestosome 1/p62 are the main components of Mallory–Denk bodies (MDB) specific for alcoholic steatohepatitis and NASH [89]. The major molecular processes involved in MDB formation are associated with an elevated ratio of keratin 8 to keratin 18 [90]. Imbalance in KRT8/KRT18 expression occurs in alcoholic liver disease (ALD) and promotes MDB formation [91]. Harada et al. proved that KRT18 overexpression inhibited MDB formation and protected against inflammatory infiltration [92]. Betterman et al. showed that KRT18 deficiency in hepatocytes led to steatosis, which increased with age, and ultimately to steatohepatitis [93]. This study indicated that a PUFA-rich diet with an LA/ALA ratio of 5:1 could disturb hepatocellular type I and type II keratin homeostasis and promote liver steatosis.

HSPA9 is a member of the heat shock protein 70 (Hsp70) family [94]. This protein expression was downregulated in the livers of animals from the 5:1 group when compared to the SFA group. HSPA9 plays a significant role in chaperoning misfolded proteins, mitochondrial import and assistance in iron-sulfur cluster (ISC) biogenesis [95,96]. HSPA9 is overexpressed in numerous types of tumors and is involved in carcinogenesis and progression processes [97], including hepatocellular carcinoma metastasis. Reduced HSPA9 expression in immortalized cells causes growth arrest. Thus, HSPA9 is a potential target for HCC cancer therapy [98]. The decreased HSPA9 expression in the group of animals fed the high-PUFA diet with an LA/ALA ratio of 5:1 could be caused by n–3 PUFA administration, which partially prevented liver damage caused by excessive amounts of FAs.

## 5. Conclusions

Our study revealed differential regulation of metabolic processes in the livers of mice fed high-fat diets with qualitative differences in fatty acids composition. Moreover, the results showed that high-fat diets increased, to varying degrees, plasma biochemical parameters, including total cholesterol and glucose concentrations. The replacement of saturated fatty acids with PUFAs resulted in many changes in the liver protein expression profiles. These changes could result in alterations in various processes, e.g., increased fatty acid β-oxidation, reduced glucose synthesis and glycolysis or redox balance changes. These distinct changes in liver metabolism could potentially explain differences in mortality and metabolic disorders disclosed in epidemiological studies on the type and amount of consumed fat.

The current study could not distinguish which dietary LA/ALA ratio was less destructive for the liver. Nevertheless, the results provided strong evidence for the different influence of low and high LA/ALA dietary ratios on liver metabolism, as two proteins were upregulated in the 5:1 group (OAT, LACTB2) and four proteins were downregulated (APOA1, CES1D, GALK1, ALDH1L1) in comparison to the 14:1 group. Thus, these results led us to the conclusion that the dietary recommendation of replacing SFAs with PUFAs is too general and should be investigated in more detail.

Solving the problem of an improperly balanced diet in terms of fatty acids is a matter of alleviating adverse metabolic changes, including overweight, obesity, hyperglycemia and hyperlipidemia, which is particularly important in the 21st century. Appropriate recommendations regarding dietary fat content and quality can prevent the development of chronic diseases causing disability, death and increased health care expenditure.

## Figures and Tables

**Table 1:** Gas Chromatography Parameters.

Group	Components	[g]	LA/ALA	% SFA	% PUFA	% MUFA
SFA	Labofeed H	790	1.41	76.87	11.04	12.09
	virgin coconut oil	200				
	pumpkin seed oil	10				
14:1	Labofeed H	790	13.76	1.68	82.21	16.10
	pumpkin seed oil	210				
5:1	Labofeed H	790	5.00	9.91	79.69	10.40
	sunflower seed oil	80				
	pumpkin seed oil	65				
	avocado oil	20				
	virgin coconut oil	20				
	hemp seed oil	15				
	corn oil	10				

LA/ALA, linoleic acid (LA, 18:2n–6) to α-linolenic acid (ALA, 18:3n–3) ratio; SFA, saturated fatty acids; MUFA, monounsaturated fatty acids; PUFA, polyunsaturated fatty acids.

**Table 2:** Plasma biochemical parameters after three-month diets—the standard (STD), the experimental diets (SFA, 14:1 and 5:1)—expressed in arithmetic mean values (SD) (8 per group).

Parameter	STD	SFA	14:1	5:1
albumin [g/L]	33.34 (1.51)	32.39 (1.74)	34.01 (1.91)	31.54 (1.36)
total protein [g/L]	53.61 (4.19)	57.09 (7.38)	59.01 (8.48)	49.80 (2.21)
total bilirubin [μmol/L]	1.58 (0.65)	1.65 (0.34)	1.09 (0.54)	1.49 (0.59)
alanine aminotransferase [U/L]	41.00 (17.78)	36.25 (10.31)	38.25 (16.00)	32.75 (6.02)
aspartate aminotransferase [U/L]	138.13 (62.66)	93.00 (21.84)	106.50 (47.88)	109.13 (65.12)
cholinesterase [U/L]	8219.75 (2215.14)	6931.88 (981.76)	6659.50 (1134.28)	6563.75 (488.00)
lipase [U/L]	13.66 (1.15)	21.04 (2.95)	23.39 (6.48)	18.49 (2.97)
iron [μmol/L]	19.99 (4.52)	26.33 (5.22)	26.14 (4.50)	25.84 (4.42)
glucose [mmol/L]	**5.29 ^C,E^ (1.66)**	**12.01 ^D^ (1.32)**	**10.60 ^D^ (1.68)**	**11.40 ^F^ (1.44)**
total cholesterol [mmol/L]	**2.83 ^A,C^ (0.31)**	**3.98 ^B^ (0.48)**	**4.44 ^D^ (0.63)**	3.60 (0.58)
high-density lipoprotein-cholesterol [mmol/L]	2.79 (0.33)	3.81 (0.64)	4.12 (0.86)	3.53 (0.58)
low-density lipoprotein-cholesterol [mmol/L]	0.12 (0.13)	0.39 (0.18)	0.37 (0.27)	0.31 (0.20)
triacylglycerols [mmol/L]	0.91 (0.28)	1.39 (0.38)	1.13 (0.17)	1.17 (0.31)

Significant differences between means were determined using the statistical model (1). Results were considered significant at ^AB^—*p* < 0.05; ^CD^—*p* < 0.01; ^EF^—*p* < 0.001 and highlighted in bold.

**Table 3:** Differentially expressed protein spots between the standard (STD) and the high-fat diet groups (SFA, 14:1, 5:1) in mouse liver.

Spot Number (SSP)	Protein Name	Accession Number UniProtKB/NCBI	Gene Symbol	*MASCOT Score*	E-Value	Sequence Coverage	Peptides Matched /Peptides Searched	Theoretical pI/MW (Da)	Fold Change					
									SFA/STD	14:1/STD	5:1/STD	14:1/SFA	5:1/SFA	5:1/14:1
0106	Regucalcin	Q64374/NP_033086.1	*Rgn*	188	1.1e-14	61%	16/28	5.15/ 33,899	1.13	1.34 (**)	1.20	1.19	1.07	0.90
0403	ATP synthase subunit beta, mitochondrial	P56480/NP_058054.2	*Atp5f1b*	346	1.7e-30	70%	32/49	5.19/ 56,265	1.39	1.29	0.78	0.92	0.56 (**)	0.61 (*)
0410	ATP synthase subunit beta, mitochondrial	P56480/NP_058054.2	*Atp5f1b*	298	1.1e-25	59%	28/37	5.19/ 56,265	0.79	0.72	1.58	0.91	2.00	2.21 (*)
1001	Lactoylglutathione lyase	Q9CPU0/NP_001107032.1	*Glo1*	72	3.90e-03	30%	5/13	5.24/ 20,967	1.12	1.48 (***)	1.24	1.32 (**)	1.11	0.84
1002	Apolipoprotein A-I	Q00623/NP_033822.2	*Apoa1*	168	1.1e-12	48%	18/46	5.51/ 30,597	0.65 (*)	0.96	0.44 (***)	1.47 (*)	0.67	0.46 (***)
1201	Galactokinase	Q9R0N0/NP_058601.2	*Galk1*	95	2.1e-05	33%	11/40	5.17/ 42,668	1.36 (**)	1.36 (**)	1.05	1.00	0.78 (*)	0.77 (*)
1309	Keratin, type I cytoskeletal 18	P05784/NP_034794.2	*Krt18*	256	1.7e-21	66%	27/61	5.22/ 47,509	0.86	0.88	0.73 (**)	1.03	0.86	0.83
1507	60 kDa heat shock protein, mitochondrial	P63038/NP_034607.3	*Hspd1*	213	3.4e-17	42%	22/38	5.91/ 61,088	1.15	0.94	0.82	0.82	0.71 (**)	0.87
2102	Prohibitin	P67778/NP_032857.1	*Phb*	184	2.7e-14	65%	13/26	5.57/ 29,859	0.80 (***)	0.78 (***)	0.79 (***)	0.98	1.00	1.02
2104	NADH dehydrogenase [ubiquinone] iron-sulfur protein 3, mitochondrial	Q9DCT2/NP_080964.1	*Ndufs3*	155	2.1e-11	47%	11/20	6.67/ 30,302	0.89	0.85 (*)	0.81 (**)	0.95	0.91	0.96
3010	Ferritin light chain 1	P29391/NP_034370.2	*Ftl1*	108	1.1e-06	53%	8/23	5.66/ 20,847	1.16	0.87	0.79	0.75 (*)	0.69 (**)	0.92
3103	Ketohexokinase	P97328/NP_032465.2	*Khk*	61	4.90e-02	26%	5/14	5.59/ 33,300	1.44 (**)	1.29	1.32	0.89	0.91	1.02
3403	S-adenosylmethionine synthase isoform type-1	Q91X83/NP_598414.1	*Mat1a*	179	8.5e-14	50%	20/43	5.51/ 44,051	0.66 (*)	0.53 (***)	0.62 (***)	0.80 (*)	0.93	1.16
3505	Sulfite oxidase, mitochondrial	Q8R086/NP_776094.2	*Suox*	215	2.1e-17	45%	16/26	6.07/ 61,231	1.15	1.20 (**)	1.09	1.04	0.94	0.90
3605	Albumin	P07724/NP_033784.2	*Alb*	288	1.1e-24	54%	29/55	5.75/ 70,700	0.45 (**)	0.47 (**)	0.41 (**)	1.06	0.93	0.87
3614	Stress-70 protein, mitochondrial	P38647/NP_034611.2	*Hspa9*	245	2.1e-20	48%	29/46	5.81/ 73,701	1.07	1.01	0.73	0.94	0.69 (*)	0.73
3806	Cytosolic 10-formyltetrahydrofolate dehydrogenase	Q8R0Y6/NP_081682.1	*Aldh1l1*	114	2.7e-07	21%	13/23	5.64/ 99,502	1.57 (*)	1.88 (***)	1.37	1.19	0.87	0.73 (*)
4101	Indolethylamine N-methyltransferase	P40936/NP_033375.1	*Inmt*	113	3.4e-07	51%	10/23	5.75/ 30,068	0.88	0.87	0.82 (*)	0.98	0.93	0.94
4102	ester hydrolase C11orf54 homolog	Q91V76/NP_001186413.1	N/A	120	6.7e-08	46%	13/40	5.86/ 35,430	1.30 (**)	1.35 (**)	1.51 (***)	1.04	1.16	1.12
4301	Ornithine aminotransferase, mitochondrial	P29758/NP_058674.1	*Oat*	205	2.1e-16	53%	20/36	6.19/ 48,723	0.62 (***)	0.60 (***)	0.68 (***)	0.97	1.09	1.13 (*)
5101	Endoribonuclease LACTB2	Q99KR3/NP_663356.1	*Lactb2*	86	1.80e-04	26%	8/17	5.89/ 33,019	1.09	1.00	1.26 (*)	0.92	1.16	1.27 (*)
5110	Peroxiredoxin-6	O08709/NP_031479.1	*Prdx6*	189	8.5e-15	72%	15/33	5.71/ 24,969	1.17	1.45 (***)	1.44 (***)	1.25	1.24	0.99
5806	Pyruvate carboxylase, mitochondrial	Q05920/NP_001156418.1	*Pc*	101	5.3e-06	15%	13/22	6.25/ 130,344	1.27	1.19	0.91	0.94	0.71 (**)	0.76
6003	Haloacid dehalogenase-like hydrolase domain–containing protein 3	Q9CYW4/NP_077219.1	*Hdhd3*	117	1.3e-07	35%	10/24	6.31/ 28,237	1.01	1.21 (*)	1.11	1.20 (*)	1.10	0.92
6305	Isovaleryl-CoA dehydrogenase, mitochondrial	Q9JHI5/NP_062800.1	*Ivd*	95	2.2e-05	34%	12/38	8.53/ 46,695	0.74 (***)	1.02	0.97	1.39 (***)	1.32	0.95
6501	Carboxylesterase 1D	Q8VCT4/NP_444430.2	*Ces1d*	124	2.7e-08	33%	15/31	6.17/ 62,034	1.12	1.34 (*)	0.81	1.20	0.73	0.60 (**)
6606	Triokinase/FMN cyclase	Q8VC30/NP_663471.1	*Tkfc*	249	8.5e-21	55%	27/40	6.44/ 59,938	1.45 (***)	1.28	1.15	0.88	0.79 (*)	0.90
7104	Carbonic anhydrase 3	P16015/NP_031632.2	*Ca3*	231	5.3e-19	81%	16/34	6.89/ 29,633	1.13	1.41 (*)	1.56 (***)	1.25 (*)	1.39 (*)	1.11
7205	Arginase-1	Q61176/NP_031508.1	*Arg1*	225	2.1e-18	72%	19/52	6.51/ 34,957	0.95	0.73 (***)	0.86	0.77 (**)	0.91	1.18
7407	Hydroxymethylglutaryl-CoA synthase, mitochondrial	P54869/NP_032282.2	*Hmgcs2*	97	1.4e-05	25%	13/32	8.65/ 57,300	1.39 (**)	0.92	1.02	0.66 (*)	0.73	1.10
7706	long-chain–fatty-acid--CoA ligase 1	P41216/NP_032007.2	*Acsl1*	116	1.7e-07	24%	14/31	6.81/ 78,928	0.78	0.65 (**)	0.75	0.83	0.97	1.16
8011	Glutathione S-transferase P 1	P19157/NP_038569.1	*Gstp1*	86	1.90e-04	48%	13/53	7.68/ 23,765	0.86	1.05	1.24	1.22	1.44 (*)	1.18
8101	Carbonic anhydrase 3	P16015/NP_031632.2	*Ca3*	237	1.3e-19	81%	18/81	6.89/ 29,633	0.97	1.14	1.33 (*)	1.18	1.37 (**)	1.16
8106	Glycerol-3-phosphate dehydrogenase [NAD(+)], cytoplasmic	P13707/NP_034401.1	*Gpd1*	119	8.5e-08	40%	12/32	6.75/ 38,176	1.42 (**)	1.06	1.42 (**)	0.75 (*)	1.00	1.33 (*)
8203	Acetyl-CoA acetyltransferase, cytosolic	Q8CAY6/NP_033364.2	*Acat2*	83	3.40e-04	27%	8/30	7.16/ 41,727	1.00	1.30 (*)	1.55 (**)	1.31	1.55 (**)	1.19
8216	Serine--pyruvate aminotransferase, mitochondrial	O35423/NP_057911.2	*Agxt*	92	4.1e-05	33%	11/44	8.37/ 46,282	1.20	1.24	1.93 (*)	1.03	1.61	1.56
8406	Homogentisate 1,2-dioxygenase	O09173/NP_038575.2	*Hgd*	103	3.4e-06	28%	11/28	6.86/ 50,726	1.18	0.62 (*)	0.77	0.52 (***)	0.65 (**)	1.25

The results show the highest identification values of proteins from an average of three biological replicates. The statistically significant (*p* < 0.05) value of the average intensity of the SFA, 14:1, 5:1 groups compared to the STD group, as well as the 14:1 and 5:1 groups compared to the SFA, and the 5:1 group compared to the 14:1 group. The significance of differences estimated using the model (1). *—*p* < 0.05; **—*p* < 0.01; ***—*p* < 0.001.

## Supplementary Materials

The following are available online at https://www.mdpi.com/article/10.3390/nu13051678/s1, Table S1: List of immunoblot antibodies; Table S2: List of reference and target genes investigated in the analysis; Figure S1a,b: Western blot replicates as uncropped images; Figure S2: Amplicon products lengths; Figure S3a,b: Amplicon products melting curves.
