# Supplementary material for: Effects of Three-Month Administration of High-Saturated Fat Diet and High-Polyunsaturated Fat Diets with Different Linoleic Acid (LA, C18:2n–6) to α-Linolenic Acid (ALA, C18:3n–3) Ratio on the Mouse Liver Proteome
Source: Nutrients. 2021 May 15;13(5):1678. doi: 10.3390/nu13051678 (PMC8156955; doi:10.3390/nu13051678)
Supplement: Supplementary file 1 [file nutrients-13-01678-s001.zip › Table S1.pdf]

Table S1. List of immunoblot antibodies.

Target Protein	UniProtKB Accession Number	Gene Symbol	Molecular Weight	Dilution	Host Species	Conjugate	Company	Code Number
Primary antibody								
ornithine aminotransferase	P29758	Oat	49 kDa	1:500	mouse	monoclonal	Santa Cruz Biotechnology, Inc	sc-376050
GAPDH	P00355	GAPDH	36 kDa	1:10000	rabbit	polyclonal	Abcam plc	ab190304
peroxiredoxin 6	P30041	PRDX6	25 kDa	1:400	mouse	monoclonal	Santa Cruz Biotechnology, Inc.	sc-101522
Secondary antibody								
m-IgGκ BP-HRP					mouse	IgGκ light chain binding protein conjugated to horseradish peroxidase (HRP)	Santa Cruz Biotechnology, Inc.	sc-516102
Peroxidase AffiniPure Goat Anti-Rabbit IgG (H+L)					goat	polyclonal, horseradish peroxidase (HRP) conjugates	Jackson ImmunoResearch Europe Ltd.	111-035-003
