# Supplementary material for: Effects of Three-Month Administration of High-Saturated Fat Diet and High-Polyunsaturated Fat Diets with Different Linoleic Acid (LA, C18:2n–6) to α-Linolenic Acid (ALA, C18:3n–3) Ratio on the Mouse Liver Proteome
Source: Nutrients. 2021 May 15;13(5):1678. doi: 10.3390/nu13051678 (PMC8156955; doi:10.3390/nu13051678)
Supplement: Supplementary file 1 [file nutrients-13-01678-s001.zip › Table S2.pdf]

Table S2. List of reference and target genes investigated in the analysis.

Gene Symbol	Gene Name	Sequence Accession Number	Primer Sequence (5' → 3')	Start	Stop	Amplicon Length [bp]	Exon Junction
B2m	beta-2 microglobulin	NM_009735.3	Fvd: CCCGCCTCACATTGAAATCC	204	223	208	397/398 (reverse primer)
			Rvs: TCACATGTCTCGATCCCAGT	411	392		
Ppia	peptidylprolyl isomerase A	NM_008907.2	Fvd: GTCTCCTTCGAGCTGTTTGC	102	121	150	113/114 (forward primer)
			Rvs: GCGTGTAAAGTCACCACCCT	251	232		
Gak	cyclin G associated kinase	NM_153569.2	Fvd: TTGTAAAGGACAGCTGGTGGAG	507	528	134	514/515 (forward primer)
			Rvs: TGATGGGTGGTTTCTGCCTG	640	621		
Oat	ornithine aminotransferase	NM_016978.2	Fvd: GGCGGTTTATACCCTGTGTCT	1026	1046	99	1040/1041 (starter Fvd)
			Rvs: TAGTGGGTTTCCGCCGTATG	1124	1105		
Prdx6	peroxiredoxin 6	NM_001303408.1	Fvd: TTTCCTGGGAGATTCCTGCTGA	175	196	63	189/190 (starter Fvd)
			Rvs: GTTGGCTTGGCCTTGAAGTTAG	237	216		
