# Supplementary figures and images for: Effects of Three-Month Administration of High-Saturated Fat Diet and High-Polyunsaturated Fat Diets with Different Linoleic Acid (LA, C18:2n–6) to α-Linolenic Acid (ALA, C18:3n–3) Ratio on the Mouse Liver Proteome
Source: Nutrients. 2021 May 15;13(5):1678. doi: 10.3390/nu13051678 (PMC8156955; doi:10.3390/nu13051678)

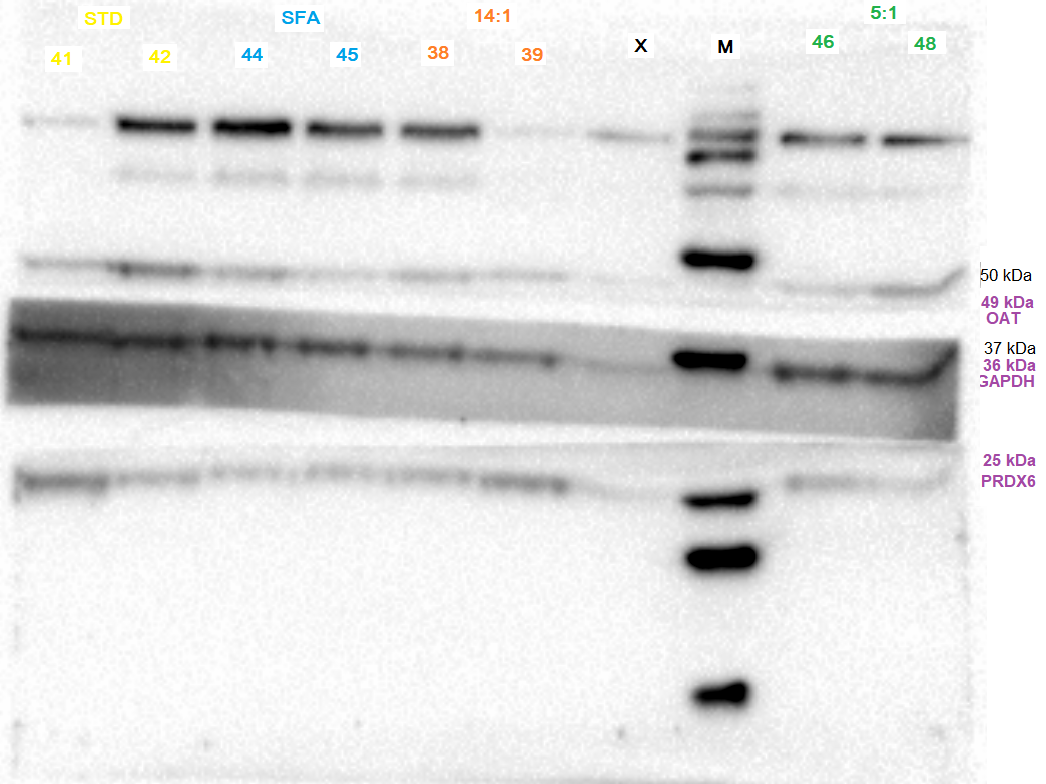

Supplement: Supplementary file 1 [file nutrients-13-01678-s001.zip › Figure S1a.tif]

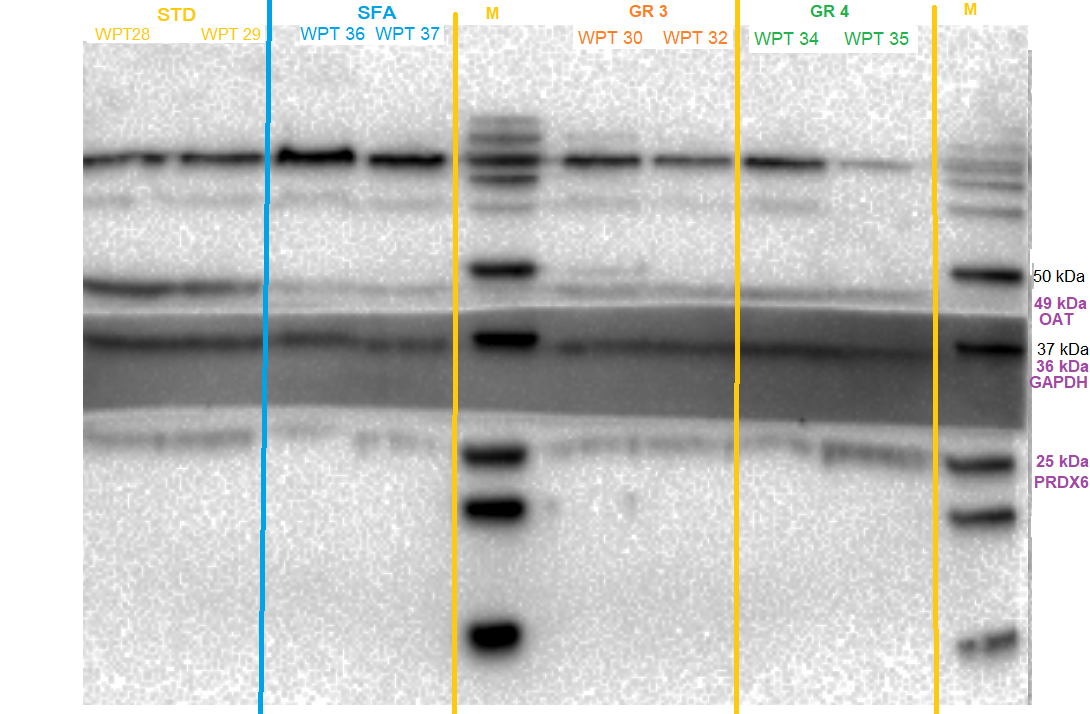

Supplement: Supplementary file 1 [file nutrients-13-01678-s001.zip › Figure S1b.png]

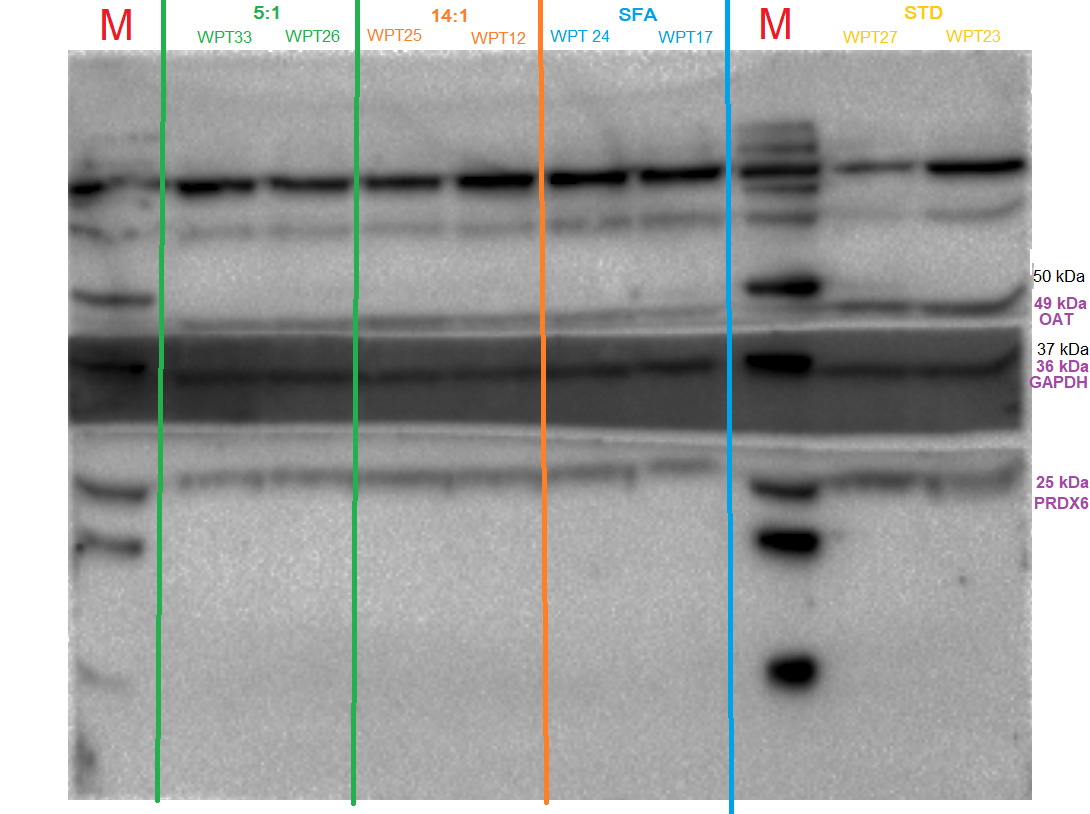

Supplement: Supplementary file 1 [file nutrients-13-01678-s001.zip › Figure S1c.png]

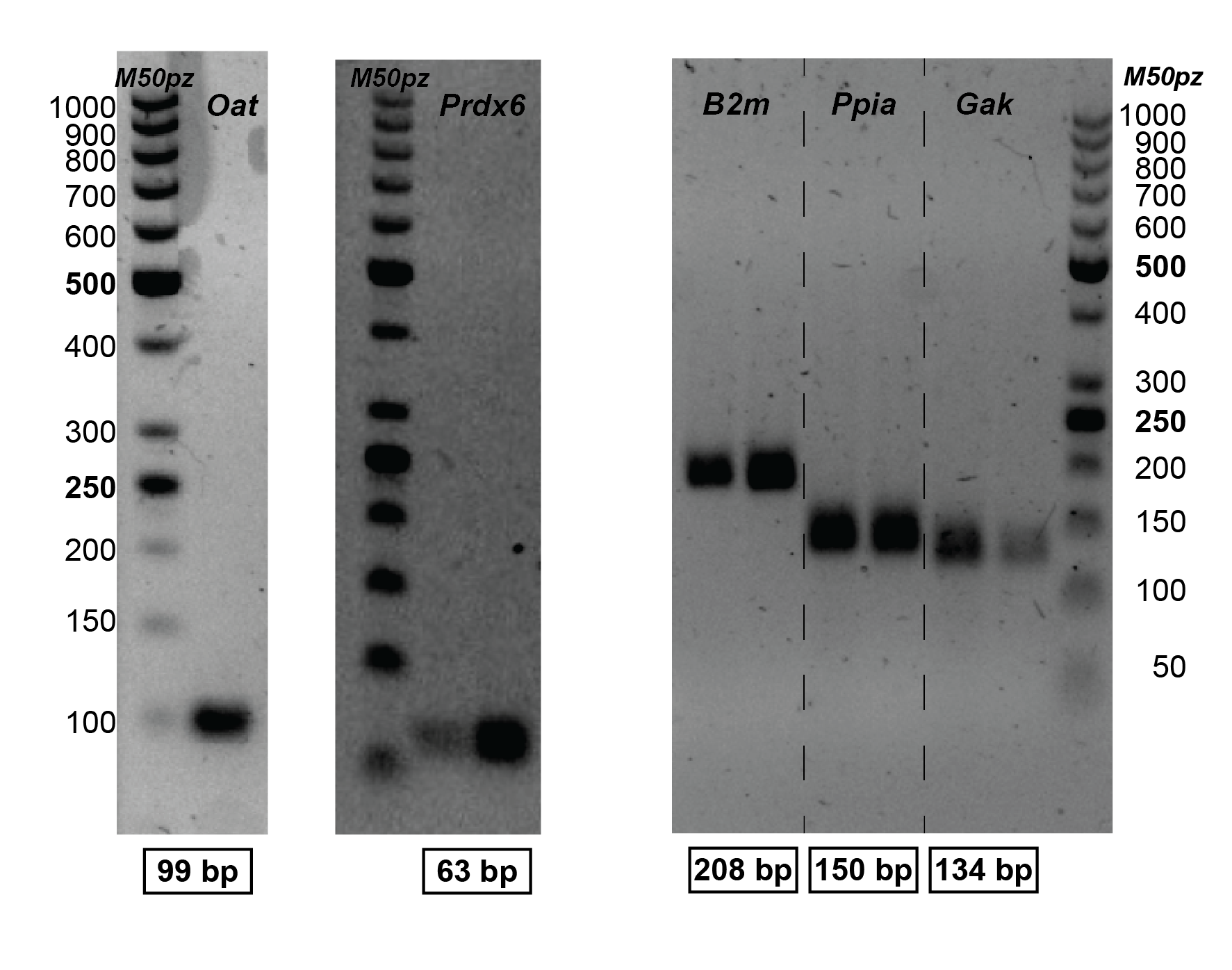

Supplement: Supplementary file 1 [file nutrients-13-01678-s001.zip › Figure S2.png]

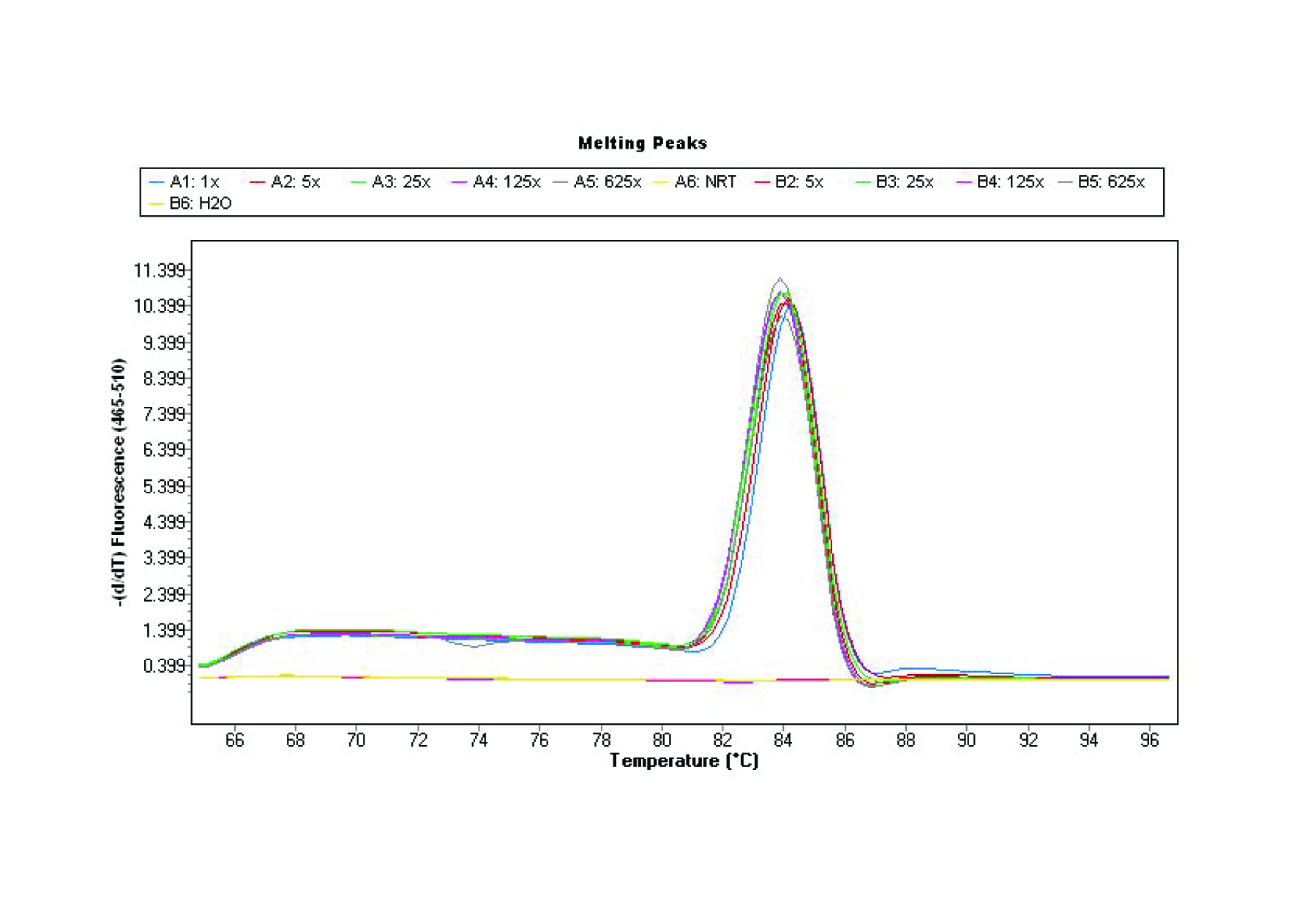

Supplement: Supplementary file 1 [file nutrients-13-01678-s001.zip › Figure S3a.tif]

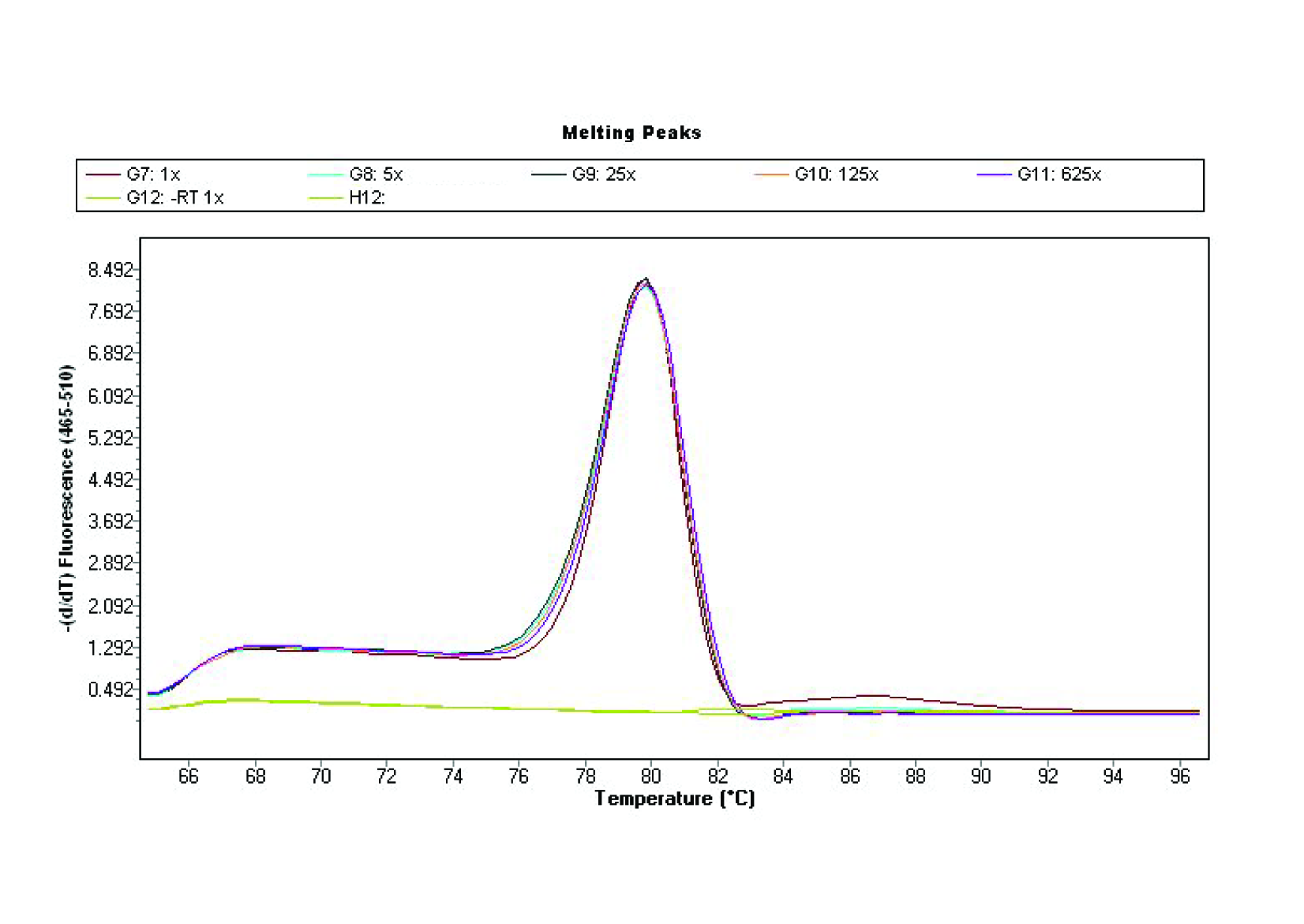

Supplement: Supplementary file 1 [file nutrients-13-01678-s001.zip › Figure S3b.tif]
